# Mitochondrial transplantation for delirium superimposed on dementia: from pathogenic mechanisms to clinical translation challenges

**DOI:** 10.3389/fnagi.2026.1885815

**Published:** 2026-08-07

**Authors:** Weiwei Hu, Yue He, Qiaoling Peng, Fang Sun

**Affiliations:** Department of Geriatric Neurology, Jinling Hospital, Affiliated Hospital of Medical School, Nanjing University, Nanjing, China

**Keywords:** delirium superimposed on dementia, mitochondrial dysfunction, mitochondrial transplantation, neurodegenerative diseases, neuroprotection

## Abstract

Delirium Superimposed on Dementia (DSD) is a common neuropsychiatric disorder in hospitalized elderly populations with poor prognosis, which can accelerate cognitive decline and increase mortality. Despite its clinical significance, there is a lack of effective therapeutic methods in clinical practice. Mitochondrial dysfunction, characterized by impaired energy metabolism, excessive reactive oxygen species (ROS) production and neuroinflammation activation, has been suggested as a potentially critical pathogenic link in DSD. As an emerging organelle-based therapy, mitochondrial transplantation (MTT) restores cellular energy homeostasis and mitigates oxidative stress by delivering functional mitochondria into damaged cells, thus holding promising potential as a future strategy for DSD treatment. This review systematically summarizes the hypothesized pathological role of mitochondrial dysfunction in DSD and the technical system of MTT, including mitochondrial isolation, purification, preservation and delivery strategies. We further elaborate on the plausible neuroprotective mechanisms of MTT and its preclinical evidence in neurodegenerative disease models relevant to, but distinct from, DSD. Additionally, we comprehensively analyze the technical, immunological and clinical challenges of MTT in DSD treatment, and propose targeted solutions and future research directions. This review constructs a theoretical framework for the hypothetical translation of MTT from basic research to clinical application in DSD, and provides novel insights for the development of future etiological therapies for this devastating disorder.

## 1 Introduction

Delirium superimposed on dementia (DSD) is a complex and under-recognized neuropsychiatric disorder characterized by the acute onset of delirium in patients with pre-existing dementia, and it has become a major clinical challenge in geriatric care. The disorder is highly prevalent in hospitalized elderly populations, with a reported prevalence of approximately 22–89% (Burnand et al., 2026), and its occurrence is closely associated with exacerbated cognitive decline, increased rehospitalization rates and elevated mortality in dementia patients (Morandi and Bellelli, 2020; Lu et al., 2025). Despite its severe clinical consequences, current therapeutic strategies for DSD remain largely symptomatic, focusing on risk factor modification and symptom control, and there is a critical lack of etiological treatments targeting the underlying pathological mechanisms. Currently, the management of DSD prioritizes nonpharmacological interventions as first-line strategies, including optimizing environmental cues, ensuring adequate hydration and nutrition, and implementing structured sleep–wake cycle protocols to reduce cognitive confusion (Fong et al., 2015). Pharmacological treatments are reserved for severe cases with distressing symptoms, where low-dose atypical antipsychotics like quetiapine or risperidone may be cautiously prescribed to alleviate agitation, though their use is limited by potential adverse effects on cardiovascular and neurological function in vulnerable elderly populations (Morandi et al., 2017). Emerging research also explores the role of adjuvant therapies, such as melatonin receptor agonists to regulate sleep disturbances or cholinesterase inhibitors to enhance cognitive stability (Lawlor et al., 2020), though further clinical trials are needed to confirm their efficacy and safety specifically for DSD patients.

The pathogenesis of DSD is intricate and involves a complex interplay of neuroinflammation, blood–brain barrier (BBB) disruption and mitochondrial dysfunction. Accumulating evidence has demonstrated that mitochondrial dysfunction may serve as a significant pathological contributor and promising therapeutic target in both delirium and dementia, which are highly relevant and overlapping clinical syndromes (Bugiani, 2021; Lee et al., 2024). As the primary cellular energy factories, mitochondria are critical for sustaining neuronal survival and function. Emerging evidence suggests that mitochondrial dysfunction—marked by impaired energy metabolism, excessive reactive oxygen species (ROS) generation, and heightened neuroinflammation—may contribute to neuronal damage and cognitive impairment linked to DSD. Given these potential associations, mitochondria have emerged as a promising therapeutic target for a range of neurodegenerative and neurovascular diseases (Khan et al., 2023). Furthermore, neurodegenerative diseases such as Alzheimer’s disease (AD) and Parkinson’s disease (PD) are closely linked to the development of delirium (Zheng et al., 2023; Ebersbach et al., 2019). However, mitochondrial-targeted interventions remain largely unexplored and represent a critical therapeutic gap for DSD. From these findings, mitochondrial transplantation may emerge as a potential future therapeutic strategy for DSD.

Mitochondrial transplantation (MTT), an innovative organelle-based therapeutic strategy, involves delivering healthy, functional mitochondria from donor cells or tissues into damaged recipient cells to restore mitochondrial function, alleviate apoptosis and suppress inflammatory responses (Riou et al., 2025). Preclinical studies have demonstrated that MTT exerts robust neuroprotective effects in neurodegenerative disease models (Nitzan et al., 2019; Wal et al., 2023), including restoring neuronal energy metabolism, reducing oxidative stress and improving cognitive function, which implies that MTT could represent a potential etiological treatment strategy for conditions with pathological features analogous to those of DSD. However, no direct preclinical studies of MTT in models of delirium or DSD currently exist. The application of MTT in DSD remains largely hypothetical and unexplored: the specific role of MTT in reversing DSD-related pathological changes, the optimal technical pathway for MTT in central nervous system targeting, and the clinical translation challenges of MTT in elderly DSD patients have not been systematically elucidated.

**Figure 1:**
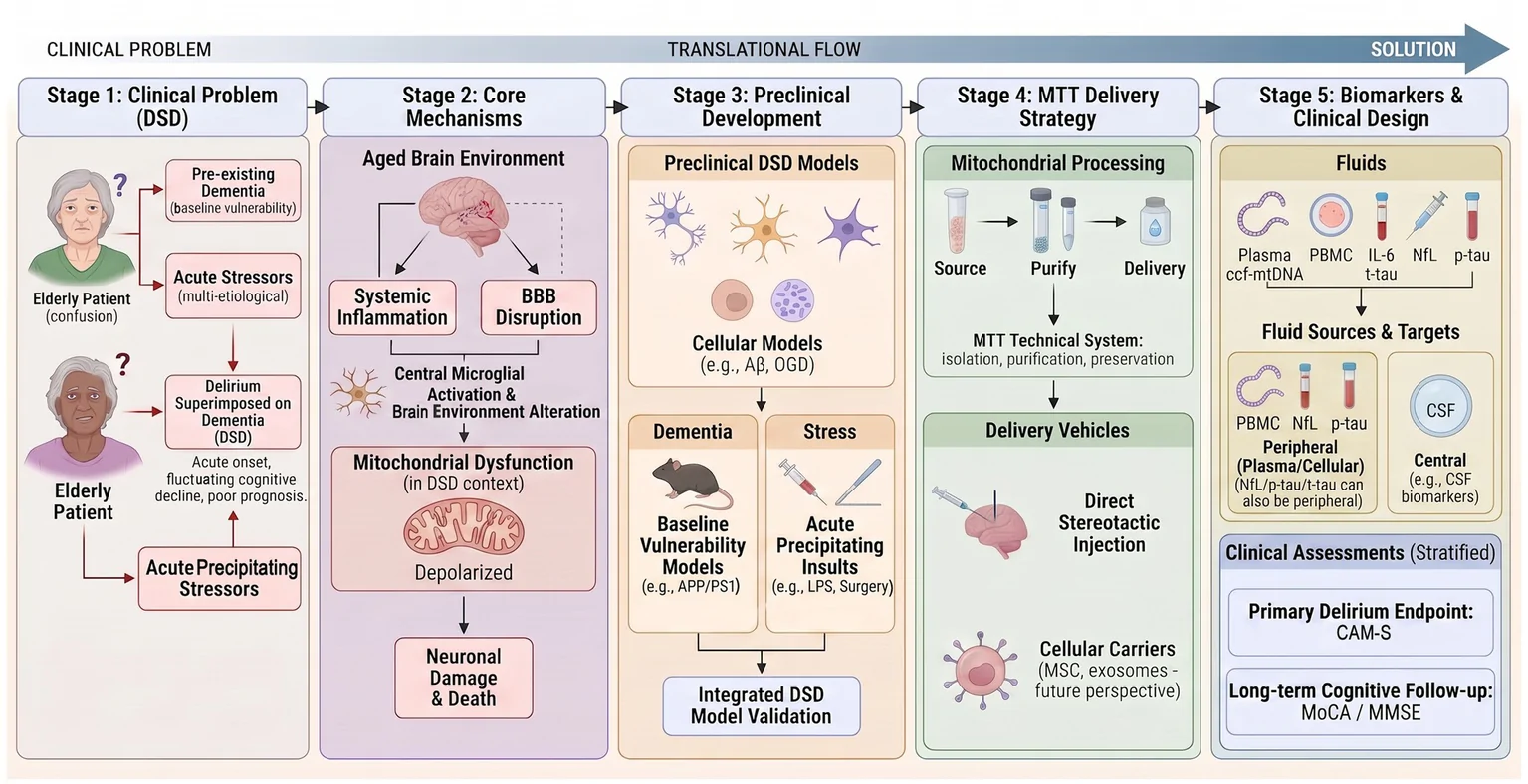
Translational framework for delirium superimposed on dementia (DSD): from mechanisms to mitochondrial transplantation therapy (MTT). Stage 1 (clinical problem): acute stressors trigger delirium on pre-existing dementia. Stage 2 (core mechanisms): inflammation and BBB disruption cause microglial activation, mitochondrial depolarization, and neuronal death. Stage 3 (preclinical development): validation of DSD models by overlaying baseline vulnerability with acute insults. Stage 4 (MTT delivery strategy): mitochondrial processing and delivery methods. Stage 5 (biomarkers & clinical design): stratified translation scheme leveraging fluid biomarkers and clinical endpoints. A*β*, amyloid-beta; APP/PS1, amyloid precursor protein/presenilin 1; BBB, blood–brain barrier; CAM-S, Confusion Assessment Method-Severity; ccf-mtDNA, circulating cell-free mitochondrial DNA; CSF, cerebrospinal fluid; DSD, delirium superimposed on dementia; IL-6, interleukin-6; LPS, lipopolysaccharide; MMSE, Mini-Mental State Examination; MoCA, Montreal Cognitive Assessment; MTT, mitochondrial transplantation therapy; NfL, neurofilament light chain; OGD, oxygen–glucose deprivation; p-tau, phosphorylated tau; t-tau, total tau.

To the best of our knowledge, this review represents one of the first attempts to systematically explore the potential therapeutic value and associated challenges of MTT in the context of DSD. Herein, we outline a plausible pathological framework linking mitochondrial dysfunction to DSD pathogenesis, and describe the hypothesized neuroprotective mechanisms of MTT. We summarize recent advances in MTT technical systems and preclinical studies conducted in dementia and age-related cognitive impairment models that share potential pathological overlaps with DSD. We further discuss key technical, immunological, and clinical challenges that may need to be addressed to advance MTT for DSD treatment, and propose targeted future research directions to support potential clinical translation. The aim of this review is to provide a preliminary theoretical framework for a hypothetical MTT-based etiological therapy for DSD, and to encourage further basic and clinical research in this emerging field, with the ultimate goal of improving the prognosis and quality of life of individuals affected by DSD (Figure 1).

## 2 Pathophysiologic mechanisms and mitochondrial dysfunction in DSD

### 2.1 Overview of the pathophysiology of DSD

The core pathologic mechanisms of DSD involve multiple complex molecular bases, particularly neuroinflammation, blood–brain barrier disruption, and mitochondrial dysfunction. The interaction of these mechanisms not only exacerbates neuronal damage but also accelerates the process of cognitive decline.

Neuroinflammation plays a pivotal role in the pathogenesis of DSD. It activates microglia and induces them to release a variety of cytokines, such as interleukin-6 (IL-6) and tumor necrosis factor-*α* (TNF-α), which can trigger and maintain a state of neuroinflammation, leading to neuronal damage and dysfunction (Su et al., 2023; Lu et al., 2025). In elderly patients with dementia, the onset of delirium is often accompanied by acute physiological stress responses, which further exacerbate neuroinflammation and form a vicious cycle where neuroinflammation and stress responses mutually reinforce each other, leading to the progressive decline of cognitive function (Veizi and Naharcı, 2025).

BBB disruption is also one of the important pathological mechanisms of DSD, serving as a critical downstream event of neuroinflammation and a key pathological link in DSD progression. The integrity of the BBB is crucial for maintaining the homeostasis of the central nervous system (CNS). When neuroinflammation is exacerbated, the permeability of the BBB increases, allowing harmful substances and immune cells to infiltrate into brain tissue and trigger more severe neuronal damage. BBB disruption not only aggravates the inflammatory response but also may induce metabolic disorders and neuronal necrosis (Wang et al., 2020; Lee and Funk, 2023).

Beyond these, mitochondrial dysfunction is increasingly recognized as a potentially important factor implicated in the pathogenesis of DSD. As the core of cellular energy metabolism, mitochondrial impairment leads to insufficient energy supply, enhanced oxidative stress and accelerated neuronal death (Lu et al., 2020; Liu et al., 2022; Li et al., 2025; Spina et al., 2025). In DSD, mitochondrial damage, neuroinflammation, and BBB disruption interact reciprocally, forming a complex pathological network.

### 2.2 The pathological role of mitochondrial dysfunction in DSD

Mitochondrial dysfunction is hypothesized to play a key role in the pathological mechanism of DSD, which is mainly characterized marked primarily by the loss of mitochondrial membrane potential and excessive production of ROS. This loss of membrane potential leads to the inability of mitochondria to efficiently synthesize ATP, which impairs the mitochondrial oxidative phosphorylation system and leads to a significant reduction in ATP synthesis. Neurons have an extremely high energy demand for maintaining synaptic transmission and neuronal activity; thus, insufficient ATP supply is proposed to induce neuronal dysfunction and death, and exacerbates cognitive decline in DSD patients. Meanwhile, oxidative stress, especially ROS production, leads to intracellular oxidative damage and interferes with the normal metabolic function of cells, which is particularly evident in elderly patients (Lu et al., 2020; Liu et al., 2022; Li et al., 2025; Spina et al., 2025). Lee et al. (2024) demonstrated that following low-dose lipopolysaccharide (LPS) stimulation, the hippocampus of aged mice exhibited a significant reduction in the activity of the mitochondrial respiratory chain, and this change was closely associated with cognitive impairment. In contrast, young mice were unaffected by low-dose LPS. It is concluded that in aged mice, mitochondrial dysfunction precedes neuroinflammation and cognitive decline after mild systemic injury, and they emphasized that mitochondria are a potential therapeutic target for delirium in elderly patients (Lee et al., 2024).

At the molecular level, mitochondrial dysfunction, mainly caused by mitochondrial DNA (mtDNA) damage and epigenetic abnormalities, represents a major hypothesized pathogenic element implicated in DSD and other aging-related neurodegenerative diseases (Pinto et al., 2013; Kukreja et al., 2014; Liu et al., 2022). The structural integrity of mtDNA is critical for maintaining mitochondrial homeostasis; its impairment disrupts the synthesis of essential respiratory chain proteins, thereby severely compromising cellular energy metabolism. In murine models of perioperative neurocognitive disorders, anesthesia and surgical trauma induce pronounced mtDNA methylation drift. This epigenetic shift—characterized by D-loop hypermethylation and coding-region hypomethylation in the hippocampus and prefrontal cortex—precipitates a reduction in mtDNA copy number, suppression of mitochondrial gene expression, and profound ultrastructural damage (Liu et al., 2022). Furthermore, in the context of AD, accumulating mtDNA mutations actively drive disease progression. For instance, PolgA D257A mutator mice exhibit impaired insulin-degrading enzyme (IDE)-mediated amyloid-*β* (Aβ) clearance, which exacerbates plaque deposition, cortical thinning, hippocampal atrophy, and progressive neurodegeneration (Kukreja et al., 2014). By contrast, Mito-PstI–induced neuronal mtDNA double-strand breaks alleviate plaque burden without elevating oxidative stress, implying mild mitochondrial dysfunction occurs as a consequence, not a cause, of amyloid pathology (Pinto et al., 2013).

**Figure 2:**
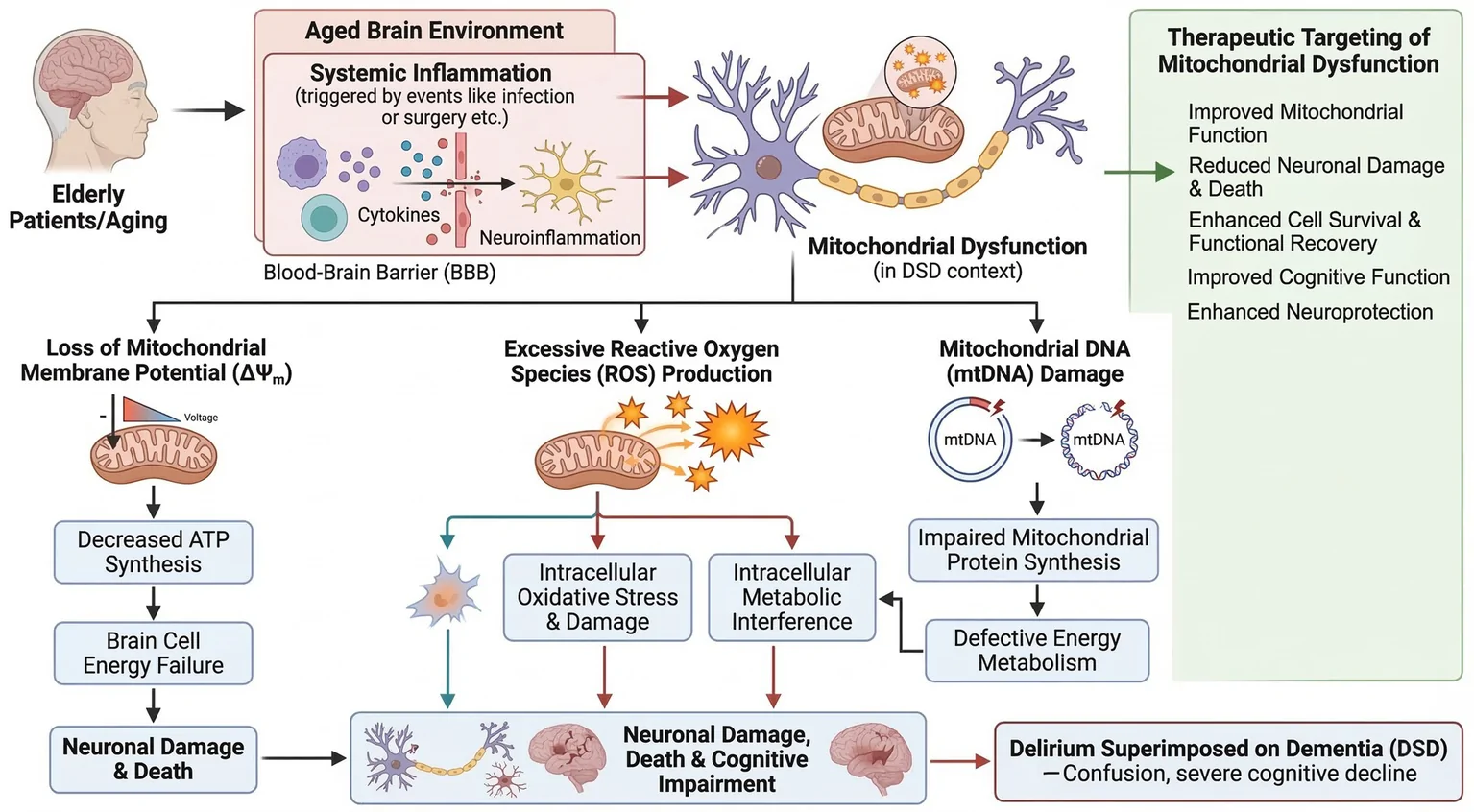
Pathogenic mechanisms of mitochondrial dysfunction in delirium superimposed on dementia (DSD) and targeted therapeutic strategies. In the aged brain, systemic inflammation disrupts the blood–brain barrier, triggering neuroinflammation and subsequent mitochondrial impairment. This dysfunction manifests through three main pathways: Loss of membrane potential causing ATP depletion, excessive ROS production inducing oxidative stress, and mtDNA damage leading to metabolic failure. These events drive neuronal death and DSD progression. Consequently, interventions restoring mitochondrial function present a promising neuroprotective approach for cognitive recovery. *ΔΨm*, mitochondrial membrane potential; ATP, adenosine triphosphate; BBB, blood–brain barrier; DSD, delirium superimposed on dementia; mtDNA, mitochondrial DNA; ROS, reactive oxygen species.

Furthermore, mitochondrial dysfunction is also closely associated with systemic inflammation in the body, especially after severe infections or surgeries, where systemic inflammatory responses trigger the BBB, leading to neuroinflammation and further mitochondrial impairment (Hua et al., 2023; Ji et al., 2023; Lee et al., 2024). Such an environment not only impairs mitochondrial function but also exacerbates the symptoms of delirium and dementia. Therefore, interventions targeting mitochondrial dysfunction may serve as an important strategy to alleviate the symptoms of DSD, providing a promising therapeutic approach. Enhanced mitochondrial function may help mitigate neuronal damage, support neuronal survival, and potentially facilitate functional recovery, which could in turn contribute to improved cognitive performance and strengthened neuroprotection (Figure 2).

## 3 Development of mitochondrial transplantation and the underlying neuroprotective mechanisms

### 3.1 Concept and technical strategies for mitochondrial transplantation

Mitochondrial transplantation represents a cutting-edge therapeutic strategy. It restores normal cellular function by transferring functional mitochondria from healthy to damaged cells. This concept stems from the recognition of the pivotal role that mitochondria play in cellular energy metabolism and overall cellular homeostasis. Mitochondria are not only the powerhouse governing cellular energy metabolism, but also participate in the regulation of vital physiological processes, including cellular metabolism, redox balance, and apoptosis. With the in-depth understanding of mitochondrial dysfunction and its pathogenic mechanisms, mitochondrial transplantation is emerging as a potential therapeutic strategy. It has demonstrated notable therapeutic potential in the treatment of mitochondrial-related disorders, neurodegenerative diseases and other related conditions (Hua et al., 2023; Ji et al., 2023; Lee et al., 2024).

Prior to mitochondrial transplantation, the extraction and purification of mitochondria represent one of the key steps for achieving efficient transplantation. Commonly used methods for mitochondrial isolation include differential centrifugation and density gradient centrifugation. These techniques can effectively isolate functionally intact mitochondria from cells for subsequent biological research.

Differential centrifugation is a classical isolation technique that separates mitochondria by subjecting samples to centrifugation at varying speeds, exploiting the differences in density and sedimentation rate between mitochondria and other cellular components, such as the nucleus and cytoplasm. This method is easy to operate and applicable to a wide range of cell types, but its limitations include potential mitochondrial damage and contamination. To enhance the purity of isolated mitochondria, density gradient centrifugation can be used in combination. During this procedure, samples are centrifuged through a density gradient medium, such as a Percoll gradient. Mitochondria form distinct bands at specific density layers, which further eliminates contamination from other cellular fractions and improves the purity and structural integrity of mitochondria (Anunciado-Koza et al., 2023).

In addition, density gradient centrifugation often requires a prolonged processing time and a relatively large sample volume. Therefore, several novel techniques have emerged in recent years, including magnetic bead affinity purification and immunopurification, which enable efficient isolation within a shorter time frame. Among these, magnetic bead affinity purification utilizes specific antibody labels to isolate mitochondria from cells via magnetic force. This approach markedly reduces interference from other cellular components and enhances the activity and integrity of mitochondria. Similarly, immunopurification labels mitochondria with specific antibodies to rapidly purify high-quality mitochondria, and it can circumvent the common drawbacks of conventional methods, such as metabolite loss and membrane disruption (Luo et al., 2021).

Quality control is of paramount importance during mitochondrial purification. Purified mitochondria must not only meet the experimental requirements in terms of quantity, but also maintain functional integrity and activity. Commonly adopted quality control criteria encompass the assessment of mitochondrial membrane potential, respiratory control ratio (RCR), and the detection of mitochondrial marker proteins. In addition, functional assays further include mitochondrial calcium uptake, respiration rate, and metabolic activity, all of which can be determined via flow cytometry or fluorescent probe-based methods (Vadovsky et al., 2024; Takegawa et al., 2025).

Mitochondrial preservation is also critical for clinical applications. Maintaining organell viability during mitochondrial transplantation represents a key factor for success. Conventional mitochondrial preservation approaches predominantly rely on cryopreservation techniques, yet such methods may lead to the loss of mitochondrial function and a decline in mitochondrial activity. The utilization of cryoprotective agents, including dimethyl sulfoxide and trehalose, can improve the survival rate of mitochondria during the cryopreservation process (Cloer et al., 2023; Roushandeh et al., 2025). In addition, the development of novel biomaterials, such as nanocapsules and biocompatible polymers, has provided new strategies for mitochondrial protection, which contribute to enhancing the stability and viability of mitochondria during *in vitro* preservation (Bodenstein et al., 2024).

Another critical determinant of successful mitochondrial transplantation is the efficient delivery of mitochondria. Particularly for the treatment of CNS disorders, penetrating the highly selective BBB remains a formidable challenge due to the relatively large size and negative surface charge of naked mitochondria (Yin et al., 2026). Current mainstream delivery technologies encompass a variety of approaches, including direct injection, cell-mediated delivery, exosome-mediated delivery, nanocarrier-mediated delivery, and cell fusion (Paliwal et al., 2018; Zhang and Miao, 2023; Cao et al., 2024; Li et al., 2024; Kim et al., 2025; Perrier et al., 2025; Von Trentini and Dmochowski, 2025). Each delivery method possesses distinct advantages and limitations, offering diverse options for clinical translation.

Direct injection is a straightforward delivery method, whereby functional mitochondria are administered directly into target cells or tissues. The primary merit of this approach lies in its operational simplicity, enabling rapid mitochondrial delivery without the need for sophisticated carriers or auxiliary equipment (Zhang and Miao, 2023). However, for brain diseases, systemic injection fails to cross the BBB; thus, invasive stereotactic intracerebral injection or non-invasive intranasal delivery—which bypasses the BBB via olfactory and trigeminal nerve pathways—are generally required (Sahu et al., 2026; Wong et al., 2026). Nevertheless, direct injection carries the risk of inducing cellular injury and reducing mitochondrial viability. Thus, careful selection of injection sites and dosages is required in clinical practice.

Cell-mediated transplantation represents an alternative approach for mitochondrial transplantation, which utilizes stem cells or other cell types as carriers to facilitate the transfer of mitochondria into target cells (Kim et al., 2025). The advantage of this strategy is that it can enhance the targeting efficiency and persistence of mitochondria. In neurological applications, carrier cells such as mesenchymal stem cells (MSCs) exhibit a natural homing capability toward inflammatory microenvironments, allowing them to cross the compromised BBB at injury sites and directly transfer mitochondria to damaged neurons. To a certain extent, cell-mediated transplantation can overcome the limitations associated with direct injection, especially in tissue regeneration and repair.

Exosome-mediated mitochondrial delivery constitutes another emerging strategy. Exosomes are small membrane-bound vesicles secreted by cells, which can efficiently transport mitochondria and preserve their stability *in vitro*. This approach achieves more efficient mitochondrial transfer through the natural intercellular signaling mechanism, while minimizing damage to target cells. Crucially, the nanoscale size and lipid bilayer structure of exosomes endow them with an inherent capacity to cross the BBB. When further surface-engineered with brain-targeting peptides (e.g., RVG peptide), exosomes can efficiently deliver mitochondrial components deep into brain tissues. Furthermore, exosomes exhibit excellent biocompatibility, rendering them suitable for clinical applications. They have demonstrated promising therapeutic outcomes in the treatment of neurodegenerative diseases and cardiovascular diseases (Cao et al., 2024; Riou et al., 2025; Von Trentini and Dmochowski, 2025; Ding et al., 2026). However, the extraction and purification of exosomes are relatively complicated, with technical bottlenecks including insufficient purity and limited yield. These issues compromise the purity and functional stability of exosomes, thereby impairing the therapeutic effect.

Nanocarrier-mediated delivery technology offers a more precise scheme for mitochondrial delivery. The use of nanoparticles as carriers can effectively enhance the targeting and bioavailability of mitochondria, optimize their distribution and accumulation *in vivo*, and consequently boost therapeutic efficacy (Li et al., 2024; Perrier et al., 2025). To overcome the BBB obstacle specifically, nanocarriers can be surface-functionalized with specific ligands or cell-penetrating peptides to actively transport mitochondria across the intact BBB via receptor-mediated transcytosis (Rajkumar et al., 2026). However, the development of nanocarriers entails overcoming multiple challenges, including the biocompatibility, safety, and in vivo stability of the materials.

Cell fusion technology exploits physiological intercellular fusion mechanisms to transfer competent mitochondria from donor cells to recipient cells. For neuropathological applications, employing BBB-permeable or neurotropic donor cells (e.g., macrophages or specific stem cell lineages) allows these cellular vehicles to traverse the endothelial barrier and subsequently fuse with compromised resident brain cells. This approach enables efficient mitochondrial transfer at the cellular level and concomitantly mitigates host alloreactivity to a certain extent (Paliwal et al., 2018; Riou et al., 2025). However, the efficiency of in vivo cell fusion is influenced by multiple complex variables, such as cell phenotype, the physical constraints imposed by the BBB, and the local tissue microenvironment, warranting further mechanistic elucidation to optimize this technique.

Importantly, the cellular uptake pattern of transplanted mitochondria within the CNS is highly dependent on the chosen delivery strategy and the localized pathological microenvironment. When direct stereotactic intracerebral injection is employed, naked mitochondria primarily scatter within the extracellular matrix and are predominantly engulfed by highly phagocytic microglia and reactive astrocytes adjacent to the injection site, with a smaller fraction internalized by neighboring damaged neurons via spontaneous endocytosis (Zhang and Miao, 2023; Sahu et al., 2026). In contrast, non-invasive intranasal delivery leverages the olfactory and trigeminal nerve pathways to bypass the BBB, allowing mitochondria to preferentially distribute along the rostral migratory stream where they are actively captured by migrating neuroblasts and enveloping glial sheets (Sahu et al., 2026; Wong et al., 2026).

For systemic routes, the cellular tropism undergoes dramatic shifts. Exosome-mediated mitochondrial delivery (particularly when surface-engineered with neurotropic peptides like RVG) shifts the internalization profile toward brain endothelial cells and neurons via receptor-mediated transcytosis and endocytosis, exploiting the natural lipid-bilayer fusion mechanisms (Cao et al., 2024; Ding et al., 2026). Meanwhile, nanocarrier-mediated delivery utilizing functionalized polymer or lipid nanoparticles can be customized to target specific cell types; TPP-coated nanoparticles enhance neuronal homing, whereas mannosylated nanoparticles redirect mitochondrial cargo predominantly to microglia (Li et al., 2024; Perrier et al., 2025). Cell-mediated transplantation (e.g., using MSCs or macrophages) takes advantage of the innate pathotropism of donor cells toward inflammatory gradients in DSD brains. These carrier cells successfully cross the compromised BBB at injury foci and subsequently transfer their mitochondrial payload into recipient astrocytes and neurons through the spontaneous formation of tunneling nanotubes (TNTs) or microvesicle shedding (Paliwal et al., 2018; Kim et al., 2025). Understanding these vehicle-specific uptake profiles is essential for tailoring MTT to rectify the multicellular dysfunction characteristic of DSD.

Overall, diverse delivery routes exist for mitochondrial transplantation, and each technical approach possesses distinct advantages and limitations. Future research should focus on optimizing these delivery strategies to improve mitochondrial survival rates, enhance BBB penetration capabilities, and boost the efficiency of functional recovery, thereby facilitating the clinical translation of mitochondrial transplantation and ultimately enhancing patient prognosis and quality of life.

### 3.2 Neuroprotective mechanisms of mitochondrial transplantation

**Figure 3:**
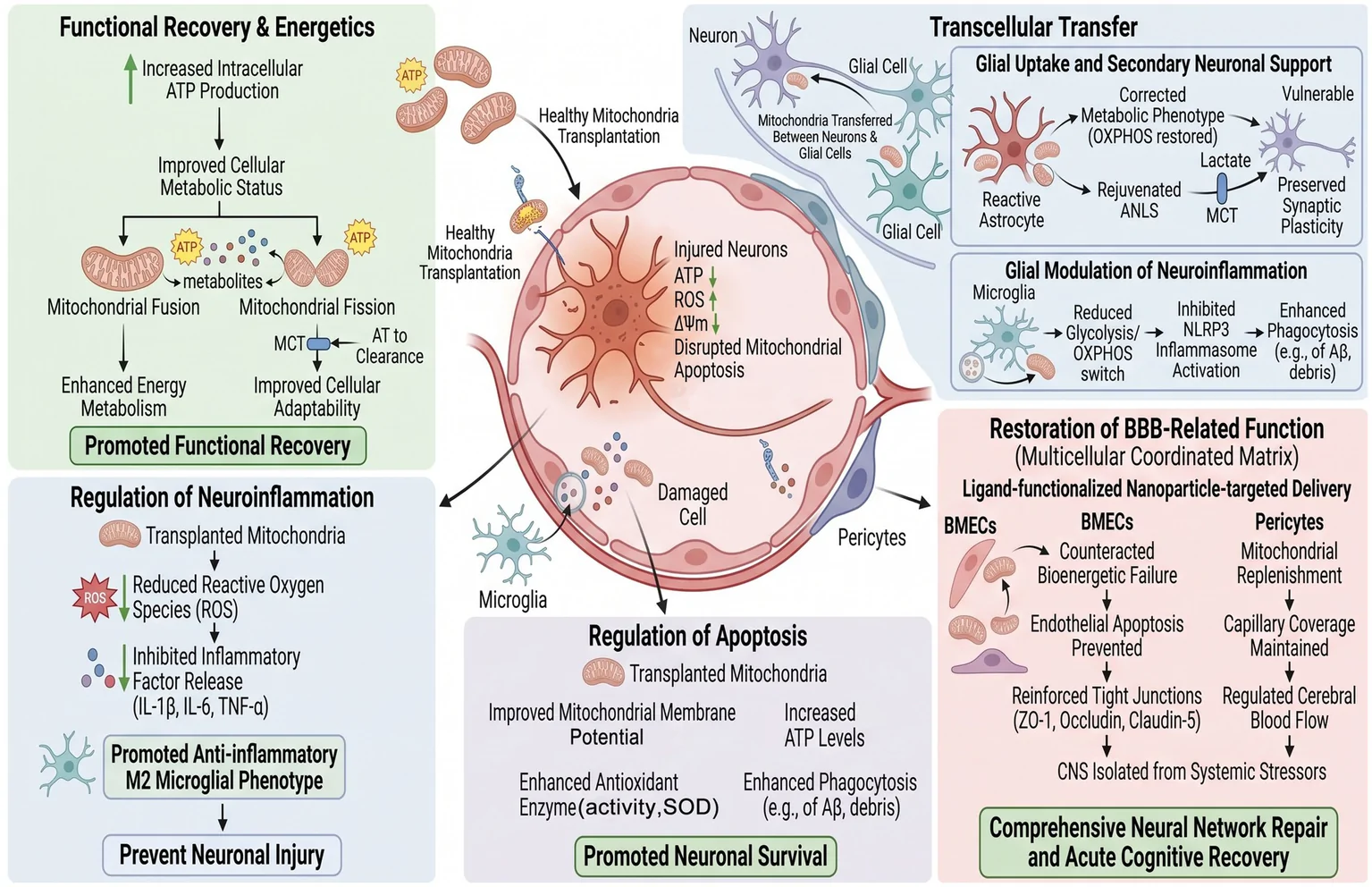
Neuroprotective mechanisms of mitochondrial transplantation. Exogenous healthy mitochondria rescue injured neurons via four synergistic pathways: (1) energetics & dynamics: increasing ATP production and restoring the fusion/fission balance; (2) neuroinflammation: reducing ROS and inflammatory factor release; (3) apoptosis: restoring ΔΨm and enhancing antioxidant defenses (e.g., SOD) to inhibit apoptotic proteins; (4) transcellular transfer: facilitating mitochondria exchange between neurons and glial cells for network-wide repair; (5) multicellular coordinated mechanisms: protecting brain microvascular endothelial cells and pericytes to reinforce tight junctions and maintain capillary coverage, thereby restoring BBB integrity and promoting network-wide repair. Aβ, amyloid-beta; ANLS, astrocyte-neuron lactate shuttle; ATP, adenosine triphosphate; BBB, blood–brain barrier; BMECs, brain microvascular endothelial cells; CNS, central nervous system; DSD, delirium superimposed on dementia; IL-1β, interleukin-1 beta; IL-6, interleukin-6; MCT: monocarboxylate transporter; MDA, malondialdehyde; MTT, mitochondrial transplantation therapy; NLRP3, NLR family pyrin domain containing 3; OXPHOS, oxidative phosphorylation; ROS, reactive oxygen species; SOD, superoxide dismutase; TNF-*α*, tumor necrosis factor-alpha; ZO-1, zonula occludens-1; ΔΨm, mitochondrial membrane potential.

Based on evidence from other neurodegenerative disease models, exogenous healthy mitochondria are suggested to exert profound neuroprotective and neurovascular restorative effects across the damaged central nervous system via five synergistic pathways: (1) Energetics and Dynamics: increasing ATP production and restoring the fusion/fission balance; (2) Neuroinflammation: reducing ROS and inflammatory factor release; (3) Apoptosis: restoring the mitochondrial membrane potential (ΔΨm) and enhancing antioxidant defenses to inhibit apoptotic proteins; (4) Transcellular Transfer: facilitating mitochondrial exchange between neurons and glial cells for network-wide repair; and (5) Multicellular Coordinated Matrix: integrating direct neuronal survival, astrocyte-driven metabolic support, microglial anti-inflammatory modulation, and neurovascular unit energetic restoration to achieve BBB sealing and comprehensive neural network repair (Figure 3).

#### 3.2.1 Energetics and dynamics

A primary hallmark of neuronal injury is bioenergetic failure. Given the exceptionally high metabolic demand of neural cells, their survival is strictly dependent on a continuous supply of ATP. Mitochondrial transplantation effectively restores the energetic deficit in damaged neurons by significantly augmenting intracellular ATP synthesis and ameliorating the overall metabolic profile. For instance, neurons subjected to oxygen and glucose deprivation (OGD) exhibit markedly elevated ATP levels and improved survival rates following mitochondrial internalization (Zhu et al., 2023). Beyond energetics, exogenous mitochondria restore the disrupted balance of mitochondrial dynamics—fusion and fission—which is critical for maintaining intracellular energy homeostasis (Chien et al., 2018). Re-established fusion facilitates inter-mitochondrial metabolite and energy exchange, whereas fission ensures the clearance of damaged organelles. By restoring this dynamic equilibrium, mitochondrial transplantation not only enhances ATP-producing capacity but also boosts cellular adaptability to microenvironmental stressors, thereby driving functional recovery (Liu et al., 2020).

#### 3.2.2 Regulation of neuroinflammation

Mitochondria are central regulators of both cellular metabolism and inflammatory responses (Ma et al., 2025). Mitochondrial dysfunction drives excessive generation of ROS, which exacerbates oxidative damage and triggers a cascade of neuroinflammatory responses, further deteriorating the pathological state of the nervous system (Yan et al., 2025). Mitochondrial transplantation directly counters this by mitigating intracellular ROS levels, dampening oxidative stress, and suppressing the release of pro-inflammatory factors. This attenuation of the inflammatory microenvironment protects neurons from secondary damage and holds significant therapeutic potential for ameliorating neuroinflammation-associated cognitive impairments (Lin et al., 2022; Kan et al., 2025).

#### 3.2.3 Inhibition of apoptosis

Exogenous mitochondria actively promote neuronal survival by intervening in apoptotic signaling cascades. Internalized healthy mitochondria stabilize the compromised ΔΨm of damaged cells and bolster intrinsic antioxidant defense systems. This is evidenced by upregulated superoxide dismutase (SOD) activity and a subsequent reduction in lipid peroxidation markers, such as malondialdehyde (MDA) and ROS (Bamshad et al., 2023; Javani et al., 2023). Together, these physiological improvements restore cellular function and inhibit the expression of pro-apoptotic proteins, effectively arresting programmed cell death and ensuring neuronal viability.

#### 3.2.4 Transcellular transfer and network repair

The therapeutic efficacy of mitochondrial transplantation extends beyond cell-autonomous mechanisms. Following neural injury, functional mitochondria undergo transcellular transfer between neurons and neighboring glial cells (Moretti et al., 2025). This intercellular exchange forms a critical network-wide rescue mechanism, facilitating the dissemination of bioenergetic support and antioxidant capacity across heterogeneous cell populations. By propagating these protective effects, transcellular mitochondrial transfer synergistically mitigates widespread inflammation and accelerates comprehensive tissue repair.

#### 3.2.5 Cellular uptake trajectories and multicellular coordinated therapeutic mechanisms

The pathological mechanism of DSD is characterized by a coordinated, multicellular network dysfunction. While MTT’s therapeutic effects in DSD remain to be fully elucidated, its neuroprotective actions could extend beyond direct neuronal rescue, potentially involving modulation of complex metabolic and inflammatory crosstalk among neurons, astrocytes, microglia, and components of the neurovascular unit.

##### 3.2.5.1 Glial uptake and secondary neuronal support

Astrocytes serve as the primary metabolic custodians of the CNS. Under different delivery strategies, particularly cell-mediated or non-targeted direct injections, a substantial fraction of exogenous mitochondria is rapidly internalized by reactive astrocytes. Instead of acting as a metabolic sink, these enriched astrocytes exhibit a corrected metabolic phenotype characterized by restored oxidative phosphorylation (OXPHOS) and enhanced lactate secretion via monocarboxylate transporters (MCTs). This metabolic reprogramming effectively rejuvenates the astrocyte-neuron lactate shuttle (ANLS), thereby providing crucial secondary energetic and trophic support to neighboring vulnerable neurons, preserving synaptic plasticity, and preventing irreversible neurodegeneration (Bélanger et al., 2011; Calì et al., 2024).

##### 3.2.5.2 Glial modulation of neuroinflammation

Microglia may be the principal innate immune cells driving the hyper-inflammatory cascade during DSD. Systemic inflammation and BBB breakdown precipitate chronic microglial priming and acute overactivation. When healthy exogenous mitochondria are internalized by microglia—frequently achieved via exosome-mediated delivery or cell fusion approaches—they actively damp down the metabolic switch from OXPHOS to glycolysis that characterizes the pro-inflammatory M1 phenotype. The restored mitochondrial energetics suppress the activation of the NLRP3 inflammasome, leading to a profound reduction in the synthesis and release of neurotoxic cytokines (such as IL-1β, IL-6, and TNF-*α*). Concurrently, MTT facilitates microglial polarization toward the neuroprotective M2 phenotype, enhancing their phagocytic capacity to clear Aβ plaques and cellular debris, thereby breaking the vicious cycle of inflammation-driven mitochondrial dysfunction (Yan et al., 2020; Lu et al., 2025).

##### 3.2.5.3 Restoration of BBB-related function

The disruption of the BBB is a critical downstream event of neuroinflammation and a principal driver of DSD pathogenesis, allowing peripheral immune cell infiltration and neurotoxic influx. Brain microvascular endothelial cells (BMECs) and pericytes constitute the structural backbone of the neurovascular unit. Delivery strategies optimized for endothelial targeting (e.g., ligand-functionalized nanoparticles) preferentially deposit their mitochondrial cargo into BMECs. Internalization of functional organelles counteracts the hypoxia- and cytokine-induced bioenergetic failure in the endothelium, prevents endothelial apoptosis, and reinforces the expression of critical tight junction proteins (such as Claudin-5, Occludin, and ZO-1) (Canfield et al., 2019; Jamieson et al., 2022; Gong et al., 2025). Furthermore, mitochondrial replenishment in pericytes preserves their structural coverage of capillaries and regulates local cerebral blood flow (Armulik et al., 2010). By repairing the structural and functional integrity of the BBB, MTT comprehensively isolates the CNS from systemic inflammatory stressors, stabilizing the cerebral microenvironment necessary for acute cognitive recovery.

In summary, MTT holds promise as a potential multicellular cytoprotective strategy for DSD. The integration of direct neuronal bioenergetic restoration, astrocyte-mediated metabolic shielding, microglia-led inflammatory dampening, and endothelial-driven barrier sealing may collectively form a synergistic therapeutic framework that could address key aspects of DSD’s complex pathophysiology.

## 4 Preclinical and clinical studies

Currently, there are no original studies specifically exploring mitochondrial transplantation in DSD or delirium animal models. The evidence discussed herein represents extrapolation from other relevant diseases. Preclinical studies of MTT across various disease models provide complementary insights indispensable for its translation to DSD. Because DSD presents as an acute cognitive fluctuation grafted upon a chronically vulnerable brain, no single experimental model can fully simulate its multiple pathogenic mechanisms. Therefore, interpreting data from diverse pathologies requires a precise translational mapping onto specific components of DSD.

Specifically, chronic neurodegenerative models—such as AD and PD—primarily inform the baseline dementia-related vulnerability, characterized by pre-existing mitochondrial depletion, progressive Aβ/tau proteinopathy, and impaired cellular clearance (Baudo, 2024; Nitzan et al., 2019; Sweetat et al., 2023; Eo et al., 2024). Ischemia/hypoxia and OGD models simulate the acute bioenergetic crisis and catastrophic ATP failure driving immediate cognitive collapse (Capobianco et al., 2024; Walker et al., 2026). Meanwhile, traumatic brain injury (TBI) and systemic endotoxemia (LPS/sepsis) models delineate pathways governing acute neuroinflammation, microglial hyper-reactivity, and BBB disruption (Lee et al., 2024; Bamshad et al., 2023; Yan et al., 2020; Lin et al., 2022). Furthermore, peripheral or systemic disease models—including osteoarthritis, idiopathic inflammatory myopathy, and ischemic heart disease—are highly relevant to DSD pathology. These models not only illustrate how peripheral inflammatory cascades propagate to the central nervous system via systemic circulation, but also rigorously validate the systemic biocompatibility, organ-homing capability, and clinical safety profiles of MTT in multi-comorbid, vulnerable hosts (Vaamonde-Garcia et al., 2025; Kim et al., 2025; Baharvand et al., 2024). Synthesizing these discrete lines of evidence through a DSD-focused framework shifts MTT from a generic organelle therapy to a mechanism-driven translational strategy (Table 1).

**Table 1:** Mechanistic mapping of MTT evidence from diverse disease models onto DSD pathophysiology.

Disease model/study category	Core evidence generated by MTT	Target DSD pathophysiological component	Translational significance for DSD management	References
AD and PD models (cellular and animal)	Restores mitochondrial membrane potential (ΔΨm) and dynamic balance Reduces Aβ plaque burden/Tau hyperphosphorylation Rescues dopaminergic/cortical cell death	Dementia Background and Vulnerability (Chronic Neurodegeneration)	MTT can repair the pre-existing fragile neural substrate of dementia, thereby enhancing baseline cognitive reserve prior to acute insults.	Baudo (2024), Nitzan et al., 2019, Sweetat et al. (2023), and Eo et al. (2024)
Ischemia/hypoxia models (oxygen–glucose deprivation—OGD)	Re-establishes OXPHOS capacity Rapidly boosts intracellular ATP synthesis Enhances cellular bioelectrical profile	Acute Energy Failure (Precipitating Acute Bioenergetic Depletion)	MTT can instantly reverse the acute bioenergetic collapse in neurons triggered by sudden systemic stressors (e.g., acute hypoxia or cerebral hypoperfusion).	Capobianco et al. (2024) and Walker et al. (2026)
Traumatic brain injury (TBI) and sepsis models	Alleviates astrogliosis and microglial hyper-activation Suppresses pro-inflammatory cytokines (IL-6, TNF-α) Promotes M2 microglial polarization	Acute Neuroinflammation and BBB Disruption	MTT can break the vicious neuroinflammatory cascade and mitigate downstream BBB hyperpermeability that drives acute delirium onset.	Bamshad et al. (2023), Yan et al. (2020), and Lin et al. (2022)
Peripheral diseases (osteoarthritis, inflammatory myopathy, cardiac disease)	Remodels hepatic/systemic metabolome Achieves safe integration without overt host alloreactivity Normalizes malate/aspartate ratio systemically	Systemic-to-CNS Inflammatory and Metabolic Axis	Illustrates how mitigating peripheral inflammation and stabilizing systemic metabolic vectors can indirectly shield the vulnerable aged brain from peripheral triggers (e.g., major surgery or severe infection).	Vaamonde-Garcia et al. (2025), Kim et al. (2025), and Baharvand et al. (2024)

AD, Alzheimer’s disease; ATP, adenosine triphosphate; Aβ, Amyloid-β; BBB, Blood–brain barrier; CNS, central nervous system; DSD, delirium superimposed on dementia; IL-6, interleukin-6; MTT, Mitochondrial transplantation therapy; OGD, oxygen–glucose deprivation; OXPHOS, oxidative phosphorylation; PD, Parkinson’s Disease; TBI, Traumatic Brain Injury; TNF-α, tumor necrosis factor-α; ΔΨm, Mitochondrial Membrane Potential.

So far, no direct preclinical studies investigating MTT have been reported in models specific to either delirium or DSD; however, studies in dementia, age-related cognitive impairment, and ischemia/hypoxia-induced neuronal damage models—which recapitulate the core pathological features of DSD (mitochondrial dysfunction, neuroinflammation, cognitive decline)—have confirmed the neuroprotective effects of MTT. Coupled with evidence from three recent clinical studies demonstrating the preliminary safety and translational potential of MTT, the need for rigorous DSD-specific models is increasingly urgent. This chapter systematically summarizes the preclinical evidence of MTT in cellular and animal models relevant to DSD, proposes strategies for constructing DSD-specific preclinical models, and lays a foundation for subsequent targeted MTT research in DSD.

### 4.1 Cellular models

In basic research on DSD, the establishment of cellular models is essential for illustrating pathogenic mechanisms and identifying potential therapeutic targets (Table 2). The human neuroblastoma cell line SH-SY5Y is widely used as a classic *in vitro* model for studying mitochondrial dysfunction and neurocognitive impairment, owing to its distinct advantages, including ease of in vitro cultivation, phenotypic stability, and the ability to recapitulate the physiological and pathological hallmarks of primary neurons (Mishra et al., 2023).

**Table 2:** Summary of common cell models for mitochondrial dysfunction in dementia and neurodegenerative diseases and the protective effects of mitochondrial transplantation.

Cell model	Induction method	Pathological characteristics	Protective effects of mitochondrial transplantation	Study
SH-SY5Y	Streptozotocin (STZ) treatment	Tau hyperphosphorylation; distorted neurites; increased ROS; induced neuronal damage	Maintained neurite density/length; reduced tau accumulation; attenuated ROS; inhibited neurotoxicity	Mishra et al. (2023)
SH-SY5Y	Aβ₁₋₄₂ treatment	Increased intracellular ROS; decreased ΔΨm; imbalanced mitochondrial dynamics; reduced ATP synthesis; activated apoptotic pathways	Receptor-mediated internalization; reduced ROS and restored ΔΨm; balanced mitochondrial dynamics; enhanced ATP synthesis; inhibited apoptosis	Baudo (2024)
SH-SY5Y	Oxygen–glucose deprivation (OGD)	Decreased ATP; increased ROS; impaired bioelectrical activity; induced apoptosis	Restored ATP synthesis; reduced ROS; improved bioelectrical profile; increased cell survival (TNTs/contact-dependent)	Capobianco et al. (2024)
SH-SY5Y	POLG p.A962T mutation	mtDNA depletion; ΔΨm depolarization; reduced ATP; decreased complex I/IV subunits; impaired respiratory complex activity	Restored mtDNA copy number and ΔΨm; increased ATP; upregulated complex I/IV subunits; improved respiratory complex activity	Hu et al. (2024)
SH-SY5Y	Okadaic acid (OA) treatment	Increased p^181^-tau; enhanced oxidative stress; decreased ΔΨm and ATP; activated mitochondrial apoptosis	Reduced p^181^-tau; suppressed ROS; restored ΔΨm, CCO activity and ATP; inhibited apoptosis via Bcl-2/Bax/caspase pathways	Zhang et al. (2020)
PC12	6-hydroxydopamine (6-OHDA) treatment	Increased ROS; induced apoptotic death; impaired neurite outgrowth	Resisted oxidative stress and apoptosis; improved viability and neurite outgrowth; restored complex I expression/dynamics; reduced oxidative DNA damage	Chang et al. (2016)
Hippocampal HT-22 cells	Erastin/RSL3 (ferroptosis induction)	Lipid peroxidation; increased mitochondrial superoxide; reduced metabolic activity; ferroptotic death	Efficient internalization; attenuated lipid peroxidation/superoxide; improved metabolic activity/survival; preserved complex I/III/V activity	Chen et al. (2023)
Primary cortical neurons (PCN)	RSL3 (ferroptosis induction)	Disrupted neuronal network; increased ferroptotic death	Exogenous mitochondrial internalization; preserved network structure; enhanced survival; reduced ferroptosis-related oxidative damage	Chen et al. (2023)

6-OHDA, 6-hydroxydopamine; ATP, adenosine triphosphate; Aβ₁₋₄₂, amyloid-β₁₋₄₂; CCO, cytochrome c oxidase; mtDNA, mitochondrial DNA; OA, okadaic acid; OGD, oxygen–glucose deprivation; PCN, primary cortical neurons; POLG, DNA polymerase subunit gamma; ROS, reactive oxygen species; STZ, streptozotocin; TNTs, tunneling nanotubes; ΔΨm, mitochondrial membrane potential.

Mitochondrial dysfunction is a central pathological event in neuronal injury and neurodegenerative diseases. As an emerging targeted intervention strategy, mitochondrial transplantation has demonstrated neuroprotective efficacy across multiple studies utilizing SH-SY5Y cell models. Three major mitochondrial dysfunction models are currently established in SH-SY5Y cells: Aβ₁₋₄₂-induced model, OGD-induced model, and DNA polymerase *γ* (POLG) mutation-mediated model (Chang et al., 2016; Zhang et al., 2020; Chen et al., 2023; Mishra et al., 2023; Baudo, 2024; Capobianco et al., 2024; Hu et al., 2024). These distinct models recapitulate the pathological hallmarks of neuronal mitochondrial damage in DSD and related neurodegenerative disorders from diverse mechanistic perspectives.

The Aβ₁₋₄₂-induced model is widely used to study AD-associated mitochondrial damage. As a common underlying disease of DSD, relevant studies on AD have provided important references for the intervention of DSD. Baudo G treated SH-SY5Y cells with Aβ₁₋₄₂ to establish an *in vitro* AD model, and applied dextran-triphenylphosphonium (Dex-TPP) functionalized mitochondria (Dex-TPP/Mt) to the damaged cells (Baudo, 2024). Their results demonstrated that Dex-TPP/Mt. efficiently entered the compromised cells via receptor-mediated endocytosis, significantly attenuating intracellular ROS levels and restoring ΔΨm. Concurrently, this intervention ameliorated mitochondrial dynamic imbalance by modulating the expression of proteins related to mitochondrial fusion and fission, and enhanced ATP synthesis in neurons. Furthermore, the study confirmed that mitochondrial transplantation exerted potent neuroprotective effects by regulating the Bax/Bcl-2 ratio and inhibiting the activation of caspase-3 and the phosphorylated c-Jun N-terminal kinase (p-JNK) signaling pathway, ultimately reducing neuronal apoptosis. These findings suggest that mitochondrial transplantation holds promise as a potential therapeutic strategy for both AD and DSD.

The OGD-induced SH-SY5Y cell model primarily simulates neuronal mitochondrial dysfunction elicited by ischemia and hypoxia, both of which are critical triggers for the onset of DSD. Research by Capobianco et al. further validated the reparative effects of mitochondrial transplantation on mitochondrial function in this model. In this study, SH-SY5Y cells were treated with OGD to successfully establish a neuronal mitochondrial dysfunction model, and the model cells showed decreased ATP levels and increased ROS production. Healthy mitochondria were subsequently transplanted using either contact or non-contact co-culture methods. The results indicated that the successfully internalized healthy mitochondria effectively restored ATP synthesis capacity in SH-SY5Y cells, suppressed ROS generation, and significantly enhanced cell viability. These findings offer *in vitro* experimental evidence that may support the potential utility of mitochondrial-targeted interventions in ischemia- and hypoxia-related neurological conditions relevant to DSD (Capobianco et al., 2024).

Besides these models, SH-SY5Y cells have been employed to simulate mitochondrial dysfunction mediated by POLG mutations, primarily recapitulating neuronal damage associated with hereditary mitochondrial diseases. This model provides a novel perspective for precision intervention strategies in DSD. Previous research (Hu et al., 2024) demonstrated that specific point mutations in the POLG gene lead to mtDNA depletion or mutagenesis, which subsequently destabilizes ΔΨm and impairs OXPHOS and ATP synthesis. Utilizing a peptide-1-mediated targeted mitochondrial transplantation strategy, researchers delivered healthy mitochondria into POLG-mutant SH-SY5Y cells. This approach effectively rescued the mutation-induced functional defects, restored mtDNA copy number, and significantly elevated ATP production levels. These findings further substantiate the feasibility and high efficiency of mitochondrial transplantation in rectifying neuronal mitochondrial dysfunction.

Cumulative evidence from various in vitro studies (Zhang et al., 2020; Mishra et al., 2023) have substantiated that, regardless of the delivery modality, the transplantation of healthy mitochondria into compromised SH-SY5Y neurons exerts robust neuroprotective effects via multiple signaling pathways. The core mechanisms underlying these effects encompass the suppression of excessive ROS generation, abrogation of mitochondria-mediated apoptotic cascades, and mitigation of oxidative stress-induced damage. These processes concurrently enhance OXPHOS activity and ATP synthesis capacity, ultimately leading to the comprehensive restoration of mitochondrial homeostasis and function.

Apart from the SH-SY5Y model, PC12 cells, hippocampal HT-22 cells, and primary cortical neurons (PCN) have also been extensively utilized in research pertaining to dementia and neurodegenerative diseases. Chang et al. demonstrated that in a PC12 injury model induced by the neurotoxin 6-hydroxydopamine (6-OHDA), mitochondrial transplantation effectively counteracted oxidative stress and apoptotic death; the surviving cells exhibited enhanced neurite outgrowth capacity (Chang et al., 2016). In another study, Chen et al. found that isolated exogenous mitochondria could be taken up by hippocampal HT-22 cells and primary cortical neurons under normal and ferroptotic conditions (Chen et al., 2023). Mitochondrial uptake can improve cell metabolic activity and enhance cell survival by reducing lipid peroxidation and mitochondrial superoxide anion production. Mechanistically, the functional integrity of mitochondrial complexes I, III, and V was found to be indispensable for this exogenous neuroprotective process. Collectively, these cell-based findings provide preliminary support for the potential reparative value of mitochondrial transplantation in mitigating neuronal dysfunction, while offering in vitro evidence that may inform the exploration of mitochondrial-targeted interventions in conditions related to DSD and other neurodegenerative disorders. These observations could lay a foundational basis for future *in vivo* studies and facilitate further investigations into potential clinical translation.

### 4.2 Animal models

In the research of DSD, mouse models of dementia are frequently employed for mechanistic exploration and therapeutic efficacy validation (Table 3). Among the widely used models are the APP/PS1 double transgenic mouse model, the Aβ₁₋₄₂-induced AD mouse model, and the 5- familial AD (5XFAD) transgenic mouse model (Zhang et al., 2015; Nitzan et al., 2019; Zhao et al., 2020; Sweetat et al., 2023).

**Table 3:** Summary of mitochondrial transplantation in dementia-related mouse models.

Mouse model	Intervention	Key mechanisms/biomarkers	Behavioral improvements	Study
APP/PS1 transgenic mice	Neural stem cell (NSC) transplantation	Mitochondrial biogenesis and mtDNA content↑; mitochondrial fission (Drp1, Fis1) and fusion (OPA1) factors↑	Significantly shortened escape latency; enhanced spatial learning and memory	Zhang et al. (2015)
Aβ₁₋₄₂-induced AD model	Intravenous (IV injection of fresh human HeLa cell-derived mitochondria)	Restored brain mitochondrial function; neuronal damage and gliosis in the hippocampus↓	Improved cognitive performance approaching wild-type levels (Fear conditioning, Y-maze, Morris water maze)	Nitzan et al. (2019)
5XFAD transgenic mice	Intravenous (IV) injection of exogenous mitochondria	Regulated synapse-associated proteins and ubiquitin-proteasome pathway; Remodeled hepatic metabolome (“liver-serum-brain” axis)	Ameliorated cognitive deficits; neuronal damage and amyloid burden↓	Sweetat et al. (2023)
Aged mice	Intravenous (IV) injection of healthy mitochondria from young mouse livers	Coexistence of heteroplasmic mtDNA; tissue ATP levels↑; ROS levels↓; macrophage phagocytic function↑	Improved learning and memory; enhanced motor endurance and innate immune function	Zhao et al. (2020)

5XFAD, 5 Familial Alzheimer’s Disease; AD, Alzheimer’s Disease; APP/PS1, amyloid precursor protein/presenilin 1; ATP, adenosine triphosphate; Aβ₁₋₄₂, Amyloid-β₁₋₄₂; Drp1, dynamin-related protein 1; Fis1, mitochondrial fission 1 protein; IV, intravenous; mtDNA, mitochondrial DNA; NSC, neural stem cell; OPA1, optic atrophy 1; ROS, reactive oxygen species.

To investigate the potential of mitochondrial interventions within these established frameworks, Zhang et al. demonstrated in APP/PS1 transgenic mice that neural stem cell (NSC) transplantation significantly improves cognitive function by promoting mitochondrial biogenesis to generate more structurally intact and functionally normal mitochondria. Morris water maze test results revealed that mice transplanted with NSC-derived mitochondria exhibited a significantly shortened escape latency to locate the hidden platform, demonstrating markedly enhanced spatial learning and memory. This effect was closely associated with the upregulated expression of mitochondrial fission-related factors (Drp1, Fis1) and fusion factor (OPA1), as well as increased mtDNA content (Zhang et al., 2015).

Complementing the findings in transgenic models, exogenous mitochondrial therapy has also shown efficacy in pharmacologically induced pathology. In the Aβ₁₋₄₂-induced AD mouse model (Nitzan et al., 2019), following the intravenous injection of freshly isolated human HeLa cell-derived mitochondria, neuronal damage and gliosis in the hippocampus were significantly attenuated, and brain mitochondrial function was effectively restored. Behavioral assessments revealed that mice in the mitochondrial transplantation group performed significantly better than the untreated group in the fear conditioning test, Y-maze test, and Morris water maze test. Their cognitive function levels approached those of normal wild-type mice, further confirming the protective effect of mitochondrial transplantation on neurocognitive function.

Expanding the scope of these therapeutic benefits to systemic mechanisms, the study by Sweetat et al. in the 5XFAD transgenic mouse model further elevated the therapeutic value of mitochondrial transplantation (Sweetat et al., 2023). The researchers observed that mitochondrial transplantation not only significantly ameliorated cognitive deficits, but also attenuated neuronal damage and amyloid burden by regulating the expression of synapse-associated proteins, ubiquitin-proteasome pathway molecules, and mitochondrial function-related factors in the hippocampus. At the same time, this intervention triggered an extensive remodeling of the hepatic metabolome, involving multiple key metabolic pathways such as glucose, glutathione, and amino acid metabolism. Moreover, aberrantly expressed metabolites in the liver could be transported to the brain via the serum, establishing a “liver-serum-brain” metabolic regulatory axis that indirectly exerts neuroprotective effects.

Beyond specific disease pathologies, the protective mechanisms of this approach also address broader senescent changes. In a model of age-related cognitive decline, Zhao et al. intravenously injected healthy mitochondria isolated from the livers of young mice into aged mice. The results revealed the coexistence of heteroplasmic mtDNA derived from both young and old donors in aged mouse tissues, accompanied by significantly elevated tissue ATP levels and reduced ROS levels. Behavioral tests confirmed that learning and memory abilities, as well as motor endurance, were notably improved. Meanwhile, the enhanced phagocytic function of macrophages suggested that mitochondrial transplantation exerts comprehensive anti-aging and cognitive-protective effects by improving energy metabolism, regulating oxidative stress, and augmenting innate immune function (Zhao et al., 2020).

While there are still no original studies specifically exploring mitochondrial transplantation for DSD, accumulating evidence from AD and age-related cognitive impairment models offers valuable mechanistic references and therapeutic implications for managing DSD. Clarifying the underlying pathological pathways, actionable therapeutic targets and intervention effects from existing work can well support further focused research into applying mitochondrial transplantation to DSD pathogenesis and treatment.

### 4.3 Clinical trials

Substantial preclinical research has confirmed that MTT exerts neuroprotective effects; clinically, this therapeutic approach has made headway in acute cerebral ischemia, inflammatory myopathies, and ischemic heart disease. Such early clinical advances have laid a solid basis for the subsequent exploration of MTT in delirium and DSD. To date, three first-in-human and early-phase clinical studies have verified the safety, clinical feasibility, and initial therapeutic efficacy of MTT across diverse patient groups, which in turn provides valuable guidance for its subsequent application in neurological conditions (Baharvand et al., 2024; Kim et al., 2025; Walker et al., 2026) (Table 4).

**Table 4:** Summary of mitochondrial transplantation in clinical trials.

Clinical model	Intervention	Key mechanisms/biomarkers	Clinical improvements	Study
Acute ischemic stroke	Intra-arterial injection of autologous mitochondria (derived from skeletal muscle) during mechanical thrombectomy	Ischemia–reperfusion injury↓; mitochondrial bioenergetics preserved	Established safety and feasibility in the human brain; achieved successful reperfusion without delayed adverse effects	Walker et al. (2026)
Acute STEMI	Intracoronary injection of autologous platelet-derived mitochondria	Mitochondrial fusion ↑; ATP production ↑; oxidative stress and apoptosis ↓	LVEF ↑; exercise capacity ↑; MACE incidence unchanged	Baharvand et al. (2024)
Refractory idiopathic inflammatory myopathy	Intravenous infusion of umbilical cord MSC–derived mitochondria (PN-101)	Inflammatory cytokines (IL-6, TNF-α)↓; malate/aspartate ratio normalized; restored ATP, and mitochondrial proteins (TOM20, SDHB)	IMACS-TISs ↑; MMT ↑; no serious AEs	Kim et al. (2025)

AEs, adverse events; ATP, adenosine triphosphate; IMACS-TISs, International Myositis Assessment and Clinical Studies Group—Total Improvement Scores; IL-6, interleukin-6; LVEF, left ventricular ejection fraction; MACE, major adverse cardiac events; MMT, manual muscle testing; MSC, mesenchymal stem cell; SDHB, succinate dehydrogenase complex iron sulfur subunit B; STEMI, ST-elevation myocardial infarction; TNF-α, tumor necrosis factor-α; TOM20, translocase of outer mitochondrial membrane 20.

In the field of acute ischemic stroke, Walker et al. (2026) conducted the first-in-human Phase 1 clinical trial of autologous mitochondrial transplantation during mechanical thrombectomy for acute ischemic stroke (AIS). The study established standardized protocols for acute isolation of viable autologous mitochondria and timely endovascular delivery within the ischemic window. Safety monitoring revealed no procedure-related adverse events, vascular complications, or systemic toxicities, with safety profiles comparable to matched controls. This trial represents the first clinical application of MTT in the human brain, demonstrating the feasibility of mitochondrial isolation and transplantation in the acute clinical setting despite logistical constraints of limited tissue and time windows.

In ischemic heart disease, a prospective, triple-blinded, randomized Phase 1 trial evaluated platelet-derived mitochondrial transplantation (PDMT) in 30 patients with acute ST-elevation myocardial infarction (STEMI) undergoing primary percutaneous coronary intervention (Baharvand et al., 2024). Intracoronary delivery of allogeneic platelet-derived mitochondria was performed following reperfusion, with comprehensive safety and efficacy monitoring. No significant differences in major adverse cardiac events, arrhythmias, or other complications were observed between treatment and control groups, confirming the safety of PDMT. The intervention significantly improved exercise capacity and trended toward enhanced left ventricular ejection fraction (LVEF) compared with standard care, establishing intracoronary mitochondrial delivery as a promising cardioprotective strategy.

Beyond specific ischemic pathologies, the protective mechanisms of this approach also address broader inflammatory and autoimmune changes. A Phase I/IIa trial by Kim et al. evaluated intravenous umbilical cord mesenchymal stem cell-derived allogeneic mitochondria (PN-101) in nine adults with refractory idiopathic inflammatory myopathy (IIM), across two dose levels (Kim et al., 2025). Over a 12-week follow-up, PN-101 demonstrated an excellent safety profile, presenting only mild, transient adverse events without severe reactions. Clinically, all patients achieved at least minimal improvement in IMACS-TIS scores. By restoring muscle mitochondrial function, mitigating inflammation, and promoting myogenesis, PN-101 exerted comprehensive therapeutic benefits. This trial validates systemic MTT as a safe and effective intervention, highlighting its potential to address extensive mitochondrial, inflammatory, and autoimmune diseases.

Across multiple clinical trials, mitochondrial transplantation has demonstrated consistent safety, feasibility, and tolerability in human participants, regardless of whether the mitochondria are autologous or allogeneic, and irrespective of the delivery route—endovascular, stereotactic, or intravenous. Early evidence of efficacy has been observed in several key domains, including improved neurological recovery, enhanced muscle function, attenuated inflammatory burden, and positive cardiac remodeling. Although direct clinical investigations of MTT in delirium and DSD have not yet been initiated, the well-established safety profiles, standardized delivery protocols, and documented mitochondrial repair in both cerebral and systemic tissues collectively lay a solid clinical groundwork for translating MTT into clinical trials targeting these conditions.

## 5 Safety and efficacy of mitochondrial transplantation

Preclinical and clinical studies in neurodegenerative diseases, cardiovascular diseases and ischemic diseases have confirmed that MTT has powerful neuroprotective and organ-protective effects, and also has good short-term safety. However, DSD patients are a uniquely vulnerable population—characterized by advanced age, pre-existing cognitive impairment, multiple comorbidities, and compromised immune/metabolic function—which imposes distinct safety requirements for MTT. This chapter systematically evaluates the preclinical safety and efficacy of MTT, highlights DSD-specific safety risks associated with the elderly population, and proposes targeted risk mitigation strategies, laying a critical foundation for the clinical application of MTT in DSD.

Mitochondrial transplantation, as an emerging therapeutic strategy, holds broad application prospects, making its preclinical safety and efficacy assessment particularly crucial. Short-term safety is a key consideration. Common side effects include local inflammation, fever, and immune reactions. These side effects are generally considered mild and reversible, indicating that patient safety can be ensured with appropriate monitoring and management (Kim et al., 2023). An autologous mitochondrial transplantation trial in patients with acute ischemic stroke reported no significant adverse events during the procedure. Safety outcomes were comparable to those in the control group without transplantation, further supporting the application of mitochondrial transplantation in acute clinical settings (Walker et al., 2026). Regarding the safety of allogeneic mitochondrial integration, preclinical studies treating osteoarthritis via intra-articular injection of allogeneic mitochondria demonstrated that transplanted mitochondria effectively integrated into joint tissues without triggering obvious immune rejection, resulting in improved joint function in subjects (Vaamonde-Garcia et al., 2025). These findings provide crucial safety data for the clinical application of mitochondrial transplantation.

While current research has demonstrated a favorable short-term safety profile, further validation through larger-scale clinical trials is still warranted. Interindividual variability among patients may give rise to divergent responses, particularly in terms of immune system function. On this basis, researchers are required to closely monitor patients’ vital signs and laboratory parameters during the implementation of mitochondrial transplantation, so as to promptly identify and manage any emerging adverse effects.

Although mitochondrial transplantation shows promising clinical prospects, long-term risks require continuous attention. Mitochondrial transplantation may increase the risk of tumorigenesis. Mitochondria play a crucial role in cellular energy metabolism, oxidative stress regulation, and cell apoptosis. When exogenous mitochondria are introduced into cells, the restoration of their function may alter the biological characteristics of cells, thereby affecting cellular proliferation and apoptosis mechanisms. In certain circumstances, such alterations may induce malignant transformation, elevating tumor incidence. Therefore, dynamic monitoring of tumorigenic risk following mitochondrial transplantation has become a necessary research direction. Despite existing studies demonstrating the anti-tumor effect of mitochondrial transplantation in certain models, its long-term impacts still need to be verified by further clinical trials (Miao et al., 2025).

Beyond the risk of tumorigenesis, mitochondrial transplantation also faces the challenge of immune rejection. Transplanted mitochondria may be recognized as foreign substances by the host immune system, triggering an immune rejection response. Such a response can compromise the success rate of transplantation and the long-term health of patients. Vaamonde-Garcia et al. demonstrated that autologous mitochondrial transplantation can markedly reduce the risk of immune rejection, as autologous mitochondria are identified as self-components by the host immune system, leading to a relatively weak immune response (Vaamonde-Garcia et al., 2025).

Besides, metabolic abnormalities represent another crucial issue that may arise during mitochondrial transplantation. Mitochondrial transplantation can improve cellular energy metabolism and restore function. In certain cases, the introduction of exogenous mitochondria may alter the metabolic pathways of host cells, resulting in metabolic disorders (Miao et al., 2025). For instance, the restoration of mitochondrial function may lead to intracellular energy surplus, which in turn induces lipid accumulation and insulin resistance—an effect that is particularly pronounced in patients with metabolic diseases such as diabetes mellitus (Chen et al., 2022).

In summary, the long-term risks associated with mitochondrial transplantation include tumorigenesis, immune rejection, and metabolic abnormalities. In-depth elucidation of these impacts requires long-term follow-up and cumulative clinical data, so as to ensure more favorable health outcomes for patients after treatment. A variety of strategies are warranted to mitigate the aforementioned risks, including personalized approaches such as the use of autologous mitochondria, combined administration of immunosuppressive regimens and the application of gene editing technologies.

The use of autologous mitochondria is regarded as one of the effective approaches to reduce immune rejection. Autologous mitochondrial transplantation can not only improve cellular energy metabolism but also enhance cell viability. Xu et al. transplanted autologous mitochondria into an ischemic brain injury model and observed significant neuroprotective effects and improvements in neurological function (Xu et al., 2024).

The integration of immunosuppressive techniques represents another widely investigated strategy. Immunosuppressants can effectively attenuate the host immune response against transplanted mitochondria and mitigate the occurrence of rejection. This approach significantly reduces the incidence of comorbidities, enhances post-transplantation cell survival, and markedly improves the overall success rate of mitochondrial transplantation (Che et al., 2022). However, since immunosuppressive therapy itself increases the risk of infection and other complications, patients must be carefully evaluated and closely monitored clinically.

In addition, the combined application of gene editing technology has opened up new avenues for personalized therapy. Through gene editing techniques such as CRISPR/Cas9, researchers can precisely target and repair gene mutations associated with mitochondrial dysfunction. This technology not only ameliorates mitochondrial function but also enhances cellular metabolic capacity, thereby boosting the success rate of transplantation (Tang et al., 2021). Meanwhile, the application of gene editing technology can also reduce the reliance on immunosuppressive therapy to a certain extent and mitigate the long-term risks for recipients.

Strategies combining autologous mitochondria with immunosuppressive or gene editing technologies are emerging as important research directions in the field of mitochondrial transplantation. These approaches not only mitigate the risks associated with mitochondrial transplantation procedures but also offer personalized therapeutic regimens for recipients. With the continuous advancement of relevant research, safer and more effective mitochondrial transplantation therapies are expected to be realized in the future.

## 6 Major challenges in mitochondrial transfer

### 6.1 Technical challenges: isolation, purification, and delivery efficiency of mitochondria

As primary cellular energy producers, mitochondria have led to the exploration of mitochondrial transplantation as a potential therapeutic strategy for conditions related to DSD. However, in this research field, the isolation, purification and delivery efficiency of mitochondria represent the pivotal technical challenges in mitochondrial transplantation studies.

First, stable isolation and purification techniques for high-quality mitochondria still require optimization. Existing mitochondrial isolation methods primarily rely on mechanical force and shear stress to homogenize tissue, followed by multiple ultracentrifugation steps for purification. These conventional approaches are typically time-consuming and may disrupt the mitochondrial double-membrane structure during processing, thereby impairing mitochondrial function. To enhance isolation efficiency and quality, emerging techniques have emerged in recent years, such as nitrogen cavitation. This method enables the rapid and efficient isolation of intact, respiratory-competent mitochondria. Not only does it streamline the complex steps of traditional techniques, but it also significantly improves the purity and activity of isolated mitochondria (Younis et al., 2023).

Second, the targeting ability of delivery systems and their capacity to cross the BBB also require further improvement. The success rate of mitochondrial transplantation largely hinges on the effective delivery of mitochondria to target cells or tissues. To this end, the application of microfluidic technology in on-chip systems has provided novel strategies for mitochondrial isolation and delivery. Microfluidic systems enable high-throughput processing, precise regulation and excellent compatibility, which greatly enhance the efficiency and accuracy of mitochondrial isolation (Ajmal et al., 2025). In addition, the development of novel delivery vectors, such as nanomaterials and biocompatible polymers, can effectively improve the *in vivo* stability and targeting of mitochondria, helping them overcome biological barriers to achieve more favorable therapeutic outcomes (Bodenstein et al., 2024; Zhou et al., 2025).

### 6.2 Immunological rejection and biocompatibility issues

Mitochondrial transplantation shows promising prospects for improving cellular function and restoring damaged tissues. However, allogeneic mitochondrial transplantation carries the risk of immunological rejection, which may significantly compromise therapeutic efficacy. Studies have demonstrated that following allogeneic mitochondrial transplantation, the recipient’s immune system may recognize these exogenous mitochondria as foreign bodies, triggering an inflammatory response and immune attack. This phenomenon has been well documented in cardiac organ transplantation; particularly, during acute rejection, the expression levels of mitochondria-associated genes in the transplanted tissue are significantly downregulated, whereas those of immune response-related genes are markedly upregulated (Romero et al., 2020). Furthermore, immune cell-mediated attack on mitochondria not only impairs mitochondrial survival but may also induce functional impairment of the transplanted tissue, exacerbating transplant rejection.

To mitigate the risk of immunological rejection, researchers are exploring various immunomodulatory strategies. These strategies primarily reduce the host immune response by regulating immune cell function and inflammatory reactions, thereby enhancing the tolerance of transplanted mitochondria. For instance, the application of stem cell therapy (e.g., mesenchymal stem cells) is recognized to suppress immune responses via the release of immunomodulatory factors and improve the survival microenvironment of transplanted tissues (Shrestha et al., 2021). At the same time, the administration of specific immunosuppressants, such as translocator protein (TSPO) ligands, can markedly inhibit the activation of B and T cells, thus reducing antibody-mediated immunological rejection (Zhang et al., 2023). In addition, researchers are investigating approaches to modify the extracellular microenvironment of mitochondria to enhance their survival capacity in the host. For example, the use of biocompatible materials (e.g., hydrogels) during transplantation can provide a favorable microenvironment for mitochondria, thereby mitigating immune cell attack and promoting mitochondrial integration (Bodenstein et al., 2024). Successful implementation of these strategies not only reduces immunological rejection and improves biocompatibility but also lays a solid foundation for the clinical translation of mitochondrial transplantation technology.

### 6.3 Standardization and barriers to clinical translation

Standardization of the manufacturing process is of paramount importance for the clinical translation of mitochondrial transplantation. Currently, substantial variations exist in the procedures for mitochondrial isolation, preparation and transplantation, alongside a lack of unified standards and protocols. Such variations not only compromise mitochondrial function and viability but also may lead to inconsistent transplant efficacy, thereby impairing the outcomes of clinical trials and the therapeutic benefits for patients. Thus, the establishment of a standardized manufacturing workflow and quality control criteria is imperative.

However, in the specific context of DSD, these procedural standardizations face a critical chronological constraint: the narrow, hyper-acute, and fluctuating clinical window of delirium. Because DSD occurs acutely in medically vulnerable older adults, the therapeutic efficacy of MTT is closely related to the velocity of organelle deployment. To successfully implement MTT within this brief window, clinical protocols must achieve unprecedented logistic acceleration. Standard operating procedures (SOPs) must be engineered to enable the ultra-rapid isolation of viable mitochondria, such as autologous from skeletal muscle/platelets or allogeneic from validated stem cell banks, microfluidic-on-chip sorting or automated magnetic bead affinity purification targeting outer mitochondrial membrane proteins (e.g., TOM20) (Vaamonde-Garcia et al., 2025). Consequently, their subsequent administration should be initiated at the earliest opportunity following the clinical identification of DSD. Delivery strategies must prioritize non-invasive, rapid brain-targeting routes. Given that systemic intravenous injection faces severe BBB restrictions and stereotactic intracerebral injection is overly invasive for highly agitated, medically unstable DSD patients, intranasal delivery emerges as the most viable translational pathway. Intranasal administration bypasses the BBB via the olfactory and trigeminal nerve pathways, allowing functional mitochondria to be delivered to the hippocampus and prefrontal cortex within minutes of isolation, perfectly aligning with the urgent clinical timeline required to abort an acute DSD (Huang et al., 2024; Shen et al., 2025). Any prolongation in processing risks missing the critical therapeutic window before irreversible, microenvironment-driven neurovascular and synaptic degradation occurs. Standardized manufacturing processes must prioritize mitochondrial storage conditions and transportation procedures to ensure that mitochondria retain their bioactivity and function during transit. Existing studies have indicated that mitochondria may sustain damage under hypothermic conditions or prolonged storage, which impairs their subsequent clinical application (Celik et al., 2025). Therefore, the development of effective storage and transportation strategies to ensure mitochondrial purity and bioactivity not only enhances therapeutic efficacy but also reduces potential adverse effects, thereby improving patient safety.

Despite the broad clinical application prospects of mitochondrial transplantation, it still faces substantial challenges in terms of clinical trial design and ethical considerations. Clinical trial design must fully account for the unique characteristics of mitochondrial transplantation, including influencing factors, expected therapeutic outcomes and potential adverse effects (Kubat, 2023). Compared with conventional therapeutic approaches, mitochondrial transplantation still lacks unified efficacy evaluation criteria, which renders the design of clinical trials complex and challenging.

Importantly, ethical considerations and surrogate consent represent a formidable barrier in DSD-specific trials, given that target subjects present a dual cognitive impairment: a baseline, pre-existing dementia compounded by an acute, fluctuating loss of decision-making capacity due to delirium. When reviewing such trials, ethics review committees are required to conduct an in-depth assessment of the sourcing, utilization and potential ethical implications of mitochondria to guarantee the regulatory compliance and ethical validity of the research. To ethically navigate consent in this vulnerable population, a rigorous proactive and multi-stage informed consent framework must be implemented. First, an “Advance Research Directive” framework should be utilized, where elderly patients with mild-to-moderate dementia proactively grant consent for future clinical trial eligibility before reaching severe cognitive decline or experiencing acute delirium. Second, when an acute DSD occurs, a legally authorized representative (LAR) or next-of-kin must provide immediate surrogate consent based on a strictly evaluated risk–benefit matrix. Third, in alignment with the fluctuating nature of delirium, a delayed “re-consent” protocol should be mandated; once the acute delirium resolves and the patient returns to their baseline cognitive state, their personal assent or consent must be sought retrospectively to maintain trial participation (Ries and Mansfield, 2021; Mozersky et al., 2023; Mahon et al., 2026).

Beyond this, participant recruitment and patient selection for clinical trials represent another critical consideration. Given that mitochondrial transplantation remains in the research phase, patient recruitment must adhere to a stringent ethical framework, with the assurance of enrolling patient cohorts that meet the study inclusion criteria. For patients with comorbidities, the risk–benefit profile of mitochondrial transplantation may be imbalanced; thus, comprehensive assessment and rigorous screening are required to ensure the scientific validity and representativeness of study findings (Dombernowsky et al., 2017; Roushandeh et al., 2025). In older DSD populations, prominent systemic comorbidities—namely frailty, active infections (e.g., urinary tract infections or pneumonia), recent major surgery, exposure to general anesthesia, and metabolic instability (e.g., severe electrolyte imbalances or glucose fluctuations)—frequently act as primary precipitating triggers for delirium while simultaneously threatening MTT safety.

Therefore, to evaluate this hypothesis safely in future clinical trial designs, eligibility criteria are theoretically proposed to stratify candidates by balancing scientific homogeneity with the profound physiological fragility of older adults.

Frailty: Elderly patients with severe frailty exhibit a significantly diminished physiological reserve and an exaggerated vulnerability to external stressors. In these patients, even minor technical instability or trace endotoxin contamination during mitochondrial preparation could trigger a catastrophic systemic inflammatory response syndrome (SIRS). Consequently, objective frailty scoring (e.g., Clinical Frailty Scale ≥ 7) should serve as an exclusion criterion in early-phase trials to balance the risk–benefit profile (Lee et al., 2020; Pienta et al., 2026).

Infection and Inflammation: Acute infections (e.g., urinary tract infections or pneumonia) are among the most common precipitating factors for DSD (Mattison, 2020). Active, uncontrolled systemic infection can exacerbate host systemic immune responses and compromise the blood–brain barrier, potentially recognizing exogenous allogeneic mitochondria as foreign pathogen-associated molecular patterns (PAMPs), thereby triggering catastrophic hyper-inflammation or transplant rejection (Peng et al., 2021; Wang et al., 2026). Eligible candidates must therefore be hemodynamically stable, with ongoing infections strictly controlled by targeted antimicrobial therapies prior to MTT.

Surgery and Anesthesia: Perioperative DSD is highly prevalent. Anesthesia and surgical trauma have been shown to induce pronounced mitochondrial DNA methylation drift, ultrastructural damage, and disturbed mitochondrial dynamics. While MTT acts as a logical rescue strategy against perioperative bioenergetic failure, the pharmacological clearance of anesthetic agents and the stability of intraoperative hemodynamics must be strictly monitored. Fluctuations in cerebral perfusion pressure during surgery could severely impair the uptake and intra-endothelial trafficking of delivered mitochondria (Liu et al., 2022; Im and Melero-Martin, 2026).

Metabolic Instability: DSD patients frequently experience acute fluctuations in blood glucose, electrolyte imbalances, and tissue hypoxia (Mattison, 2020). Because the functional survival of transplanted mitochondria strictly depends on the host cell’s baseline homeostasis, severe metabolic derangements can prematurely depolarize the transplanted mitochondria’s membrane potential, rendering the therapy ineffective and potentially accelerating recipient cell apoptosis through the leakage of cytochrome c (Chen and Chen, 2024). Consequently, strict metabolic stabilization protocols should be established as a prerequisite for trial eligibility.

The clinical translation of MTT for DSD is hindered by the synergy of general MTT challenges and DSD-specific clinical/physiological barriers. These barriers are interrelated: technical limitations (e.g., poor BBB penetration) reduce therapeutic efficacy, which complicates clinical trial design; the lack of standardized protocols leads to inconsistent results, which erodes regulatory confidence; ethical barriers for the elderly DSD population slow patient recruitment and trial completion. Addressing these barriers requires a multidisciplinary, DSD-focused approach that integrates technical development, standardization, clinical trial design, and ethical frameworks—all tailored to the unique needs of elderly, cognitively impaired DSD patients.

## 7 Challenges and future perspectives

### 7.1 Development of novel mitochondrial delivery technologies

With the in-depth understanding of the roles of mitochondria in cellular physiology and pathology, mitochondrial transplantation has attracted increasing attention as a potential therapeutic strategy. However, the clinical application of mitochondrial transplantation faces numerous challenges, particularly regarding the delivery efficiency of mitochondria and the preservation of mitochondrial bioactivity. Recent advances in nanotechnology have offered novel solutions for mitochondrial delivery. Che et al. have demonstrated that constructing nanoscale protective shells using metal–organic frameworks (MOFs) can effectively prolong mitochondrial bioactivity at room temperature for up to four weeks (Che et al., 2022). This approach not only enhances delivery efficiency but also provides a new strategy for intracellular internalization. In addition, functionalization of the MOF surface with polyethyleneimine (PEI) and the cell-penetrating peptide Tat can significantly enhance the intracellular delivery efficiency of mitochondria, further supporting the efficacy of mitochondrial transplantation in cancer cells (Zhou et al., 2025).

Research on non-invasive delivery methods represents a pivotal frontier in the development of mitochondrial transplantation technology. For instance, extracellular vesicles (EVs) have been employed as delivery carriers to successfully transport mitochondria or their functional components into target cells. This approach not only mitigates the risk of cellular damage associated with direct delivery but also enhances therapeutic targeting and efficacy (Peruzzotti-Jametti et al., 2021). Intranasal and transdermal administration also stand out as promising non-invasive routes. These pathways enable direct entry of mitochondria into the systemic circulation and efficient bypass of the BBB, thereby enhancing therapeutic efficacy (Bandiwadekar et al., 2024; Pandya et al., 2024). Bandiwadekar et al. delivered mitochondria-targeted drugs via a microneedle delivery system, an efficient non-invasive transdermal technology, which significantly improved the bioavailability of drugs in the brain and reduced side effects (Bandiwadekar et al., 2024).

Additionally, innovative research combining nanotechnology with non-invasive delivery methods is continuously advancing. By integrating mitochondria with biocompatible nanomaterials, more efficient delivery and sustained release can be achieved, leading to superior therapeutic performance.

### 7.2 Mechanistic insights and future explorations

Mitochondrial transplantation, as a novel therapeutic strategy, is gradually being applied to research on multiple neurological diseases. Mitochondria play critical roles in energy metabolism, calcium homeostasis, and cellular signal transduction in neurons, and the restoration of mitochondrial function may exert beneficial effects on the overall function of neural networks. Previous studies have demonstrated that mitochondrial transplantation can significantly alleviate neurological dysfunction induced by ischemia and oxidative stress, promoting the survival and reconnection of neurons, thereby enhancing neural network function (Norat et al., 2020). In a study on Parkinson’s disease, transplanted mitochondria were shown to effectively improve the survival rate of dopaminergic neurons and reduce neuronal inflammation. These effects are likely closely associated with the ability of mitochondria to supply intracellular energy and exert antioxidant activities (Eo et al., 2024).

Furthermore, mitochondrial transplantation may improve neural network function by facilitating interactions between neurons. Mitochondria can be transferred through intercellular channels (e.g., tunneling nanotubes), and this transfer not only provides energy support but also may enhance the overall activity of neural networks by regulating intercellular signal transmission (Jiao et al., 2024). Therefore, the restoration of mitochondrial function represents not only an improvement at the cellular level but also a potential enhancement of the overall function of neural networks. This provides novel insights and approaches for the treatment of neurodegenerative diseases caused by mitochondrial dysfunction.

Despite the promising potential exhibited by mitochondrial transplantation, numerous challenges remain in its clinical translation, including the survival rate of transplanted mitochondria, functional integration, and the ability to maintain long-term biological activity. Therefore, future research should focus on optimizing the efficacy and safety of mitochondrial transplantation to further promote its application in the restoration of neural network function.

The mechanism of mitochondrial communication between cells is a hotspot in current research, especially concerning its role in neurological diseases. Studies have shown that mitochondria are not only the main source of energy but also play a key role in intercellular signal transduction and communication. Cells achieve mitochondrial transfer through various mechanisms, including extracellular vesicles (e.g., exosomes) and TNTs, which provide new perspectives on intercellular interactions (Cao et al., 2024). Exosome-mediated mitochondrial transfer enables the intercellular delivery of energy and signals, which is particularly important in neural cells. In neurodegenerative diseases, mitochondrial transfer can significantly increase the energy supply to damaged cells and improve their functional status. For example, in animal models of multiple sclerosis, mitochondrial transfer was found to reduce neuronal damage and promote neural repair, indicating that mitochondria may play an important role in regulating immune responses and promoting cell survival (Peruzzotti-Jametti et al., 2021; Picone and Nuzzo, 2022).

In addition, mitochondrial dynamics, such as fusion and fission, are also critically involved in intercellular communication. These dynamic processes not only modulate mitochondrial function but also influence cellular physiological responses by regulating intracellular calcium concentration, redox status, and other signaling parameters. Mitochondrial fusion enhances the capacity for energy storage, whereas mitochondrial fission may facilitate cellular adaptation to environmental stressors; both processes exert beneficial effects on intercellular communication and functional recovery (Bertholet et al., 2016; Lu et al., 2020).

### 7.3 Large-scale clinical trial design

When exploring the potential of mitochondrial transplantation as a treatment for DSD, the design of large-scale clinical trials is particularly important. Multicenter, randomized controlled trials (RCTs) are a widely recognized and effective study design that can provide high-quality evidence to support the efficacy and safety of therapeutic approaches.

Such trials can be conducted in diverse medical settings and populations, thereby enhancing the external validity of the research and ensuring the generalizability of the results. According to existing literature, mitochondrial transplantation has shown promising efficacy in various diseases, improving cellular energy metabolism and restoring mitochondrial function (Kim et al., 2023). However, most of the relevant studies have focused on small-scale or single-center trials, lacking large-scale randomized controlled studies (Baharvand et al., 2024; Kim et al., 2025; Walker et al., 2026).

The implementation of multi-center RCTs necessitates extensive collaboration and coordination among various stakeholders, encompassing ethical review, patient recruitment, and data collection and analysis across all participating centers. Furthermore, SOPs must be established to ensure consistency in critical steps such as mitochondrial isolation, transplantation, and follow-up. This standardization is essential to minimize variability and enhance data reliability (Baharvand et al., 2024). Additionally, the study design should account for patient heterogeneity—including factors such as age, sex, and comorbidities—to facilitate appropriate adjustments during outcome analysis.

The establishment of a long-term follow-up and efficacy evaluation system represents a critical component for verifying the therapeutic effectiveness of mitochondrial transplantation. This system encompasses not only the assessment of short-term efficacy but also long-term outcomes, including sustained improvements in cognitive function and reductions in the incidence of delirium. Existing studies have demonstrated that mitochondrial transplantation exerts favorable neuroprotective effects in animal models; however, systematic evaluations of its long-term clinical effects remain lacking in clinical practice (Nitzan et al., 2019; Eo et al., 2024; Riou et al., 2025). To robustly evaluate the *in vivo* target engagement, biological efficacy, and therapeutic mechanisms of MTT in DSD, future trials should move beyond purely symptomatic assessments and implement a rigorous, mechanistic fluid biomarker framework. Here, we propose a hypothetical, multidimensional fluid biomarker framework that could serve as a conceptual guide for future target engagement studies of MTT in DSD. This framework is stratified across five distinct pathophysiological domains and meticulously accounts for clinical feasibility in acutely ill older adults, with each domain explicitly mapped to clinically meaningful endpoints such as delirium severity and long-term cognitive recovery.

#### 7.3.1 Multidimensional pathway-specific fluid biomarkers

A comprehensive biomarker framework for MTT evaluation should encompass five critical pathological dimensions:

##### 7.3.1.1 Mitochondrial respiration and bioenergetics

Circulating cell-free mitochondrial DNA (ccf-mtDNA) in plasma serves as a sensitive index of systemic mitochondrial stress and injury. Furthermore, the oxygen consumption rate (OCR) and extracellular acidification rate (ECAR)—indicative of mitochondrial oxidative phosphorylation and glycolysis—can be dynamically profiled via Seahorse XF analysis in freshly isolated peripheral blood mononuclear cells (PBMCs) or platelets (Jaramillo-Morales et al., 2022; Magalhaes-Novais et al., 2022; Gubert et al., 2025).

##### 7.3.1.2 ROS and oxidative stress

To evaluate whether healthy exogenous mitochondria mitigate the oxidative storm characteristic of DSD, fluid markers such as plasma MDA, 8-hydroxy-2′-deoxyguanosine (8-OHdG), and advanced oxidation protein products (AOPP) should be dynamically tracked (Wu et al., 2024; Motrenikova et al., 2025).

##### 7.3.1.3 Mitochondrial membrane potential

The preservation of ΔΨm is fundamental to arresting apoptotic cascades. Using flow cytometry on intact vital platelets or PBMCs stained with potentiometric fluorophores (e.g., JC-1 or tetramethylrhodamine ethyl ester, TMRE), investigators can evaluate whether systemic MTT successfully stabilizes host cellular membrane potential (Liang et al., 2021; Court et al., 2024; Morton et al., 2025).

##### 7.3.1.4 Systemic and neuroinflammation

Systemic acute-phase inflammation actively drives blood–brain barrier breakdown and microglial priming in DSD. Peripheral expressions of classical pro-inflammatory cytokines, including IL-6, TNF-*α*, interleukin-1 beta (IL-1β), and high-sensitivity C-reactive protein (hs-CRP), should be tracked dynamically using high-sensitivity multiplex immunoassays (Fong and Inouye, 2022; Asadizeidabadi et al., 2025).

##### 7.3.1.5 Neurodegeneration and synaptic injury

To establish whether peripheral mitochondrial recovery translates to central neuroprotection, ultra-sensitive Single Molecule Array (Simoa) technology should be utilized to quantify brain-derived proteins in plasma. Crucial targets include Neurofilament Light Chain (NfL) (reflecting axonal damage), glial fibrillary acidic protein (GFAP) (reflecting reactive astrogliosis and blood–brain barrier integrity), total tau (t-tau), phosphorylated tau (e.g., p-tau181 or p-tau217), and the Aβ42/Aβ40 ratio (monitoring the acceleration of underlying dementia pathology) (Marengoni et al., 2025).

#### 7.3.2 Feasibility constraints in acutely ill older adults

Designing biomarker protocols for DSD requires careful consideration of the clinical realities of acutely ill, fragile older populations. While cerebrospinal fluid (CSF) analysis offers the most direct reflection of central nervous system pathology, routine lumbar puncture is highly unfeasible, ethically challenging, and clinically risky in agitated, delirious, and medically unstable older adults.

Therefore, our framework prioritizes peripheral blood (plasma/serum) and urine-based assays, which possess high translational feasibility and can be seamlessly integrated into standard acute clinical workflows without adding undue distress to the patient. Advances in ultra-sensitive Simoa technology now allow the precise quantification of brain-derived proteins (such as NfL and p-tau) in peripheral blood at picogram-level concentrations, effectively bridging the gap between clinical feasibility and neurobiological accuracy (Marengoni et al., 2025). Furthermore, magnetic bead-based isolation of neural-derived extracellular vesicles (NDEVs) from plasma offers a safe, minimally invasive window into intra-neuronal mitochondrial dynamics in real time (Serpente et al., 2020).

#### 7.3.3 Linking fluid biomarkers to clinically meaningful endpoints

To achieve regulatory validation and establish clinical relevance, these fluid biomarkers must be explicitly linked to validated clinical trajectories in DSD, divided into short-term delirium severity and long-term cognitive recovery.

##### 7.3.3.1 Delirium severity and duration

Acute baseline elevations in inflammatory cytokines (IL-6, TNF-α), bioenergetics (OCR), and oxidative stress markers (MDA, 8-OHdG) are expected to strongly correlate with the peak severity and duration of delirium, objectively quantified via the Confusion Assessment Method-Severity (CAM-S) score or the Memorial Delirium Assessment Scale (MDAS) (Lin et al., 2021). Successful target engagement of MTT should manifest as a rapid, synchronized decline in these acute fluid markers, predicting a shortened duration of delirium episodes and decreased fluctuating cognitive confusion.

##### 7.3.3.2 Long-term cognitive recovery

Downstream markers of structural neurodegeneration (plasma NfL and p-tau) along with NDEV complex protein recovery should serve as key predictors of long-term cognitive trajectories. Attenuation of the post-acute rise in plasma NfL levels following MTT intervention would provide robust biochemical evidence of neuroprotection, directly linking to better performance on the Mini-Mental State Examination (MMSE) and the Montreal Cognitive Assessment (MoCA) at 3-month, 6-month, and 12-month post-discharge follow-ups, as well as a lower rate of accelerated permanent cognitive decline (Mattsson et al., 2019; Moscoso et al., 2021; Maduro et al., 2025).

In summary, meticulously designed large-scale clinical trials, coupled with the establishment of a robust follow-up and evaluation system, could provide a solid scientific basis for the application of mitochondrial transplantation in DSD treatment. Future research should focus on overcoming current challenges to facilitate the clinical translation of this innovative therapeutic strategy.

### 7.4 Interdisciplinary integration and innovation

In modern medical research, interdisciplinary collaboration and integration have emerged as a crucial driving force for advancing therapeutic innovation. Particularly in the field of treating complex neurodegenerative diseases such as DSD, the combination of gene editing and stem cell technologies has provided new opportunities for the development of novel comprehensive therapeutic strategies. Gene editing technologies, typified by CRISPR-Cas9, enable precise modification of disease-associated genes to correct pathogenic mutations or regulate gene expression, demonstrating substantial potential across numerous neurodegenerative diseases (Tang et al., 2021; Bhardwaj et al., 2022). Bhardwaj et al. suggests that CRISPR-Cas9 brings new hope for the treatment of Alzheimer’s disease. By downregulating genes encoding the amyloid precursor protein (APP) or related enzymes through gene editing, the accumulation of amyloid proteins can be effectively slowed, thereby delaying disease progression (Bhardwaj et al., 2022).

Meanwhile, stem cell technology provides the capability to regenerate and repair damaged neural tissues. MSCs play a pivotal role in neuroprotection and repair; particularly in models of ischemic brain injury and other nervous system disorders, MSCs can promote the regeneration and functional recovery of neural tissues by secreting a variety of bioactive factors (Kim et al., 2020; Jiménez-Acosta et al., 2023). By combining gene editing with stem cell technology, researchers can modify stem cells to endow them with enhanced neuroprotective abilities. These modified cells can not only replace damaged neurons but also enhance their own survival and function through gene regulation (Imai et al., 2023).

The combination of gene editing and stem cell techniques is driving the evolution of personalized medicine. By analyzing the genomic information of individual patients, clinicians can formulate the most appropriate stem cell-based therapeutic regimens, thereby enhancing both the efficacy and safety of treatment. This personalized therapeutic approach has demonstrated promising prospects in clinical practice, offering tailored solutions based on the specific pathological characteristics of each patient.

Combined gene editing and stem cell techniques shed new light on DSD treatment and also underpin the creation of better-targeted therapy regimens. Further studies are therefore needed to advance the clinical translation of their synergistic effects, so as to achieve better clinical benefits for DSD patients.

Emerging biotechnologies also include the utilization of exosomes and stem cell secretions as therapeutic carriers. These biomaterials carry a variety of bioactive substances, which can act directly on the nervous system to promote neural repair and regeneration. Exosomes play a crucial role in signal transmission between neurons and effectively regulate neuronal survival and function. Current research is exploring how to optimize their therapeutic effects through engineered exosomes to achieve targeted drug delivery (Yeşilkır Baydar et al., 2024; Sheykhhasan et al., 2025; Varshney et al., 2025).

Studies utilizing exogenous mitochondrial transplantation have demonstrated that this approach not only improves cellular energy status but also provides additional protection by regulating intracellular inflammatory responses and oxidative stress. Mitochondrial transplantation can inhibit inflammatory responses by promoting the M2 polarization of microglia, thereby improving neurological function (Yan et al., 2020). These findings offer new insights for the development of innovative therapeutic strategies targeting DSD.

Collectively, continuous advancement in biotechnologies—including the synergistic application of gene editing and stem cell technologies, as well as the emerging utilization of exosomes, stem cell secretions, and exogenous mitochondrial transplantation—has endowed novel therapeutic approaches with renewed promise for the management of DSD. Future investigations are warranted to further validate the safety and efficacy of these techniques and explore their practical clinical applications, with the ultimate goal of delivering more effective therapeutic regimens for affected patients.

## 8 Conclusion

Delirium superimposed on dementia (DSD) is a highly prevalent neuropsychiatric condition among older hospitalized patients, for which targeted etiological treatments are currently lacking. Emerging evidence suggests that mitochondrial dysfunction may represent a key pathological contributor to DSD, highlighting it as a potential therapeutic target for mitochondrial transplantation. This review systematically outlines the technical framework and neuroprotective mechanisms of this approach, summarizing evidence that it markedly improves cognitive function in preclinical dementia models. The translational feasibility of this innovative strategy has been recently underscored by its progression into clinical research for ischemic stroke. Nevertheless, its translational application in DSD is hindered by several critical challenges, including an incomplete understanding of the underlying mechanisms, the lack of efficient targeted delivery vehicles, immune rejection, and broader translational barriers. Future research should prioritize the development of DSD-specific experimental models, the optimization of precise mitochondrial delivery systems, and the design of rigorous multicenter clinical trials. Through interdisciplinary integration and technological innovation, continuous advances are expected to accelerate the translation of mitochondrial transplantation from bench to bedside. Collectively, mitochondrial transplantation provides a promising novel therapeutic opportunity, with the potential to significantly improve patient prognosis and quality of life in DSD.
